# First record of the family Bemmeridae Simon, 1903 from China, with descriptions of three new species of *Damarchus* Thorell, 1891 (Arachnida, Araneae)

**DOI:** 10.3897/zookeys.1287.187795

**Published:** 2026-07-31

**Authors:** Yunjie Zhang, Kun Yu, Feng Zhang

**Affiliations:** 1 Key Laboratory of Zoological Systematics and Application, College of Life Sciences, Hebei University, Baoding, Hebei 071002, China Hebei Basic Science Center for Biotic Interaction, Hebei University Baoding China; 2 Hebei Basic Science Center for Biotic Interaction, Hebei University, Baoding, Hebei 071002, China Key Laboratory of Zoological Systematics and Application, College of Life Sciences, Hebei University Baoding China

**Keywords:** East Asia, morphology, Mygalomorphae, new record, Southwest China, taxonomy

## Abstract

The genus *Damarchus* Thorell, 1891 (Bemmeridae) represents a relatively understudied lineage of mygalomorph spiders, with previous studies documenting its distribution across several Asian countries, including India, Indonesia, Malaysia, Myanmar, Singapore, and Thailand. This study describes three new species from China, *Damarchus dai***sp. nov**., *D. erebus***sp. nov**., and *D. rutilus***sp. nov**., which represent the first formal records of the genus *Damarchus* and the family Bemmeridae for the spider fauna of China.

## Introduction

The taxonomy of the family Bemmeridae Simon, 1903 has undergone multiple revisions. The family was originally established by Simon (1903) as the tribe Bemmereae, elevated to the subfamily Bemmerinae within Nemesiidae Simon, 1899 by Raven (1985), and subsequently raised to family rank by Opatova et al. (2020). Bemmeridae currently contains four genera, from southern Africa (*Homostola* Simon, 1892 and *Spiroctenus* Simon, 1889) and Southeast Asia (*Atmetochilus* Simon, 1887 and *Damarchus* Thorell, 1891) (World Spider Catalog 2026). However, according to Kulkarni et al. (2023: fig. 5), this family is not monophyletic, as the Asian genus *Damarchus* forms a sister group to the clade comprising Barychelidae Simon, 1889 and Theraphosidae Thorell, 1869, whereas the African *Homostola pardalina* (Hewitt, 1913) is sister to a portion of Cyrtaucheniidae Simon, 1889. Moreover, the taxonomic placement of Asian bemmerid genera remains somewhat controversial. Some authors place them in Bemmeridae (or Bemmerinae; Raven 1985; Kunsete et al. 2020, 2025a, 2025b), while others treat them as incertae sedis (Opatova et al. 2020; Montes de Oca et al. 2022).

Members of *Damarchus* are small to medium mygalomorph spiders (body length ~ 10–23 mm). The burrow of these spiders consists of an open main shaft and a blind side shaft into which the spider retreats when disturbed. In most species, the entrance of the main shaft is open, while the entrance of the side shaft is closed by a pair of lateral flaps. A few species have flaps or turrets at the entrance, or can plug the side shaft with a soil pellet (Schwendinger and Hongpadharakiree 2023).

Taxonomic attention to *Damarchus* remains insufficient. Among the 11 described species of *Damarchus*, six were described before the mid-20^th^ century [*D. montanus* (Thorell, 1890), *D. workmani* Thorell, 1891, *D. oatesi* Thorell, 1895, *D. assamensis* Hirst, 1909, *D. cavernicola* Abraham, 1924, *D. bifidus* Gravely, 1935] (WSC 2026). Renewed attention to the genus in recent years has revealed five newly described species (*D. dao* Schwendinger & Hongpadharakiree, 2023, *D. lanna* Schwendinger & Hongpadharakiree, 2023, *D. pylorus* Schwendinger & Hongpadharakiree, 2023, *D. inazuma* Kunsete & Warrit, 2025, and *D. sirindhornae* Kunsete & Warrit, 2025), all limited in Thailand (Schwendinger and Hongpadharakiree 2023; Kunsete et al. 2025a, 2025b). Furthermore, the key morphological difference currently recognized between *Damarchus* and its putative sister genus *Atmetochilus* is the distribution pattern of the sternal sigilla, namely that the sigilla are discrete in the former and fused in the latter (Zonstein and Marusik 2016; Kunsete et al. 2020); however, recent studies suggest that this is a continuously varying character (Schwendinger and Hongpadharakiree 2023), which potentially challenges the generic boundaries.

Further taxonomic and systematic attention to the family Bemmeridae, particularly its Asian members, is clearly warranted. Here, we present the first report of the bemmerid fauna from China. Three new species from Yunnan Province closely match the current diagnostic characters of *Damarchus* and are consequently assigned to this genus. Moreover, given the widespread use of the mitochondrial cytochrome c oxidase subunit I (*CO I*) gene in species delimitation and systematics of mygalomorph spiders (Hamilton et al. 2011; Castalanelli et al. 2014; Ortiz and Francke 2016; Harvey et al. 2018; Hüsser 2018), we provide the *CO I* sequences as additional molecular data for these species alongside detailed morphological descriptions. This approach aims to enhance our understanding of these taxa and to offer more multidimensional data to support future research.

## Materials and methods

The specimens were collected by members of the laboratory by turning over stones or excavating burrows (Fig. 32), and all are preserved museum specimens. All specimens preserved in 75–100% ethanol were examined under a Leica M205A stereomicroscope. Photographs of genitalia were taken with an Olympus BX53 microscope equipped with a Kuy Nice CCD camera. Photographs of live specimens were taken using a Sony α7R IV with a Sony SEL90M28G macro lens. Photographs of specimens were stacked using the Helicon Focus 8 and retouched using Adobe Photoshop CC 2020. All measurements are given in millimeters, which were obtained using a Leica LAS V. 4.3 stereomicroscope. Total body length excludes chelicerae. Spination was recorded in the format: (proximal-middle-distal). Palpal measurements are given as total length (femur, patella, tibia, tarsus), with the male palpal tarsus referring to the cymbium only. Leg measurements are presented as total length (femur, patella, tibia, metatarsus, tarsus). Female genitalia were cleared using pancreatin (BBI Life Sciences). All specimens studied are deposited in the Museum of Hebei University, Baoding, China (**MHBU**).

For comparison with the previously described *Damarchus* species, we did not directly examine their specimens but instead relied mainly on the excellent illustrations from earlier works (Zonstein and Marusik 2014; Kunsete et al. 2020, 2025a, 2025b). Species concept. In the present study, we describe new species based on the morphological species concept (i.e., delimiting species based on identifiable morphological features), as in several other recent studies on *Damarchus* (Schwendinger and Hongpadharakiree 2023; Kunsete et al. 2025a, 2025b). Although considered to have certain subjectivity and limitations, the morphological species concept is still widely used in classical spider taxonomy (Bond 2012). ZooBank registration. This published work and the nomenclatural acts it contains have been registered in ZooBank. The LSID for this publication is: urn:lsid:zoobank.org:pub:F5517C98-2A62-4791-A732-06F61D303BAB.

The mitochondrial cytochrome c oxidase subunit I (*CO I*) sequences of the new species were amplified as supplementary molecular information (Table 13), aiming to provide additional species-level evidence, but were not used for species delimitation decisions in this study. Primers used for *CO I* amplification were as follows: LCO1490 (5'–GGTCAACAAATCATAAAGATATTGG–3') and HCO2198 (5'–TAAACTTCAGGGTGACCAAAAAATCA–3') (Folmer et al. 1994). PCR amplification conditions for the *CO I* were as follows: pre-denaturation for 3 min at 95 °C, 35 cycles of 30 s at 95 °C, 45s at 45 °C, 45 s at 72 °C, then a final extension at 72 °C for 7 min. PCR products were sent to Sangon Biotech Co. Ltd (Beijing, China) for sequencing. Genetic distances were analyzed in MEGA 12.0 (Kumar et al. 2024) with p-distance and K2P parameter.

### Abbreviations used in this study

Eyes: **ALE** – anterior lateral eyes; **AME** – anterior median eyes; **PLE** – posterior lateral eyes; **PME** – posterior median eyes; **MOA** – median ocular area.

Positions: **d** – dorsal; **Ds** – distal; **Md** – middle; **pd** – prodorsal; **pl** – prolateral; **Pr** – proximal; **pv** – proventral; **rd** – retrodorsal; **rl** – retrolateral; **rv** – retroventral; **v** – ventral.

Others: **Sp** – tibial spur; **PLS** – posterior lateral spinneret; **PMS** – posterior median spinneret; **XTBG** – Xishuangbanna Tropical Botanical Garden; **TPNR** – Tongbiguan Provincial Nature Reserve.

## Taxonomic account

**Family Bemmeridae Simon, 1903 (Chinese name**: 贝默蛛科)

### Genus *Damarchus* Thorell, 1891 (Chinese name. 达玛蛛属)

*Damarchus* Thorell, 1891: 14; Kunsete et al. 2020: 523.

**Type species**. *Damarchus workmani* Thorell, 1891, by original designation.

#### Damarchus dai sp. nov.

Taxon classificationAnimaliaAraneaeBemmeridae

43398D11-362E-5BB8-8B3C-88FFD3E08865

https://zoobank.org/93B91326-1C93-45DE-A163-23DB3A4D8C17

Figs 1, 2, 3, 4, 5, 6, 7, 8, 9, 10, Tables 1–4

##### Chinese name.

傣达玛蛛.

##### Type material.

***Holotype***: China • ♂; Yunnan Province, Xishuangbanna Autonomous Prefecture, Jinghong, Ake Village, 2 Apr. 2023, Z. Zhang leg.; GenBank: PX967099; KYUARA#1497. ***Paratypes***: China • 1 ♀; same data as the holotype; GenBank: PX967105; KYUARA#1498. • 1 ♂ 1 ♀; same locality as preceding, but Manmian Village, 21 Feb. 2023, Z. Zhang leg.; GenBank: PX967098, KYUARA#1493; GenBank: PX967104, KYUARA#1494.

##### Other material examined.

China • 1 ♂; Yunnan Province, Xishuangbanna Autonomous Prefecture, Menglun Town, XTBG, 15 Dec. 2022, H. Yang leg.; GenBank: PX967100 KYUARA#1492. • 1 ♀; same data as preceding, but along National Highway 213 (near XTBG), May 2023, Z. Yang leg.; GenBank: PX967101; KYUARA#1524.

##### Diagnosis.

*Damarchus dai* sp. nov. shares a similar overall appearance with *D. erebus* sp. nov., particularly in the coloration of the female legs, where the femora are distinctly darker than the remaining segments. Moreover, *D. dai* sp. nov. can be distinguished from *D. erebus* sp. nov. by: (1) the long embolus, with length ~ 1.5 × the width of the tegulum (vs almost equal to the tegulum width in *D. erebus* sp. nov.); (2) the male palpal tibia lacks a retrolateral bump (vs present in *D. erebus* sp. nov.); (3) the long spermathecal stalk, ~ 2–3 × the length of the receptacle (vs a much shorter stalk, which is similar in length to the receptacle, and distinctly everted in *D. erebus* sp. nov.).

##### Description of male.

**Figure 1.**
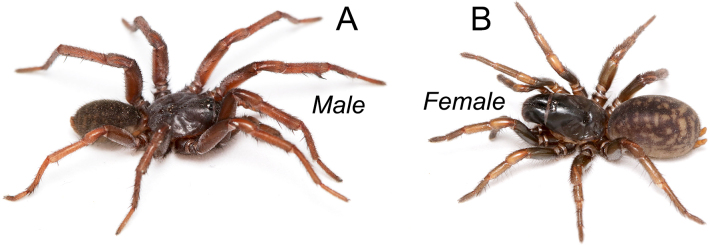
*Damarchus dai* sp. nov., living spiders from Jinghong population. **A**. Male holotype (KYUARA#1497); **B**. Female paratype (KYUARA#1498).

**Figure 2.**
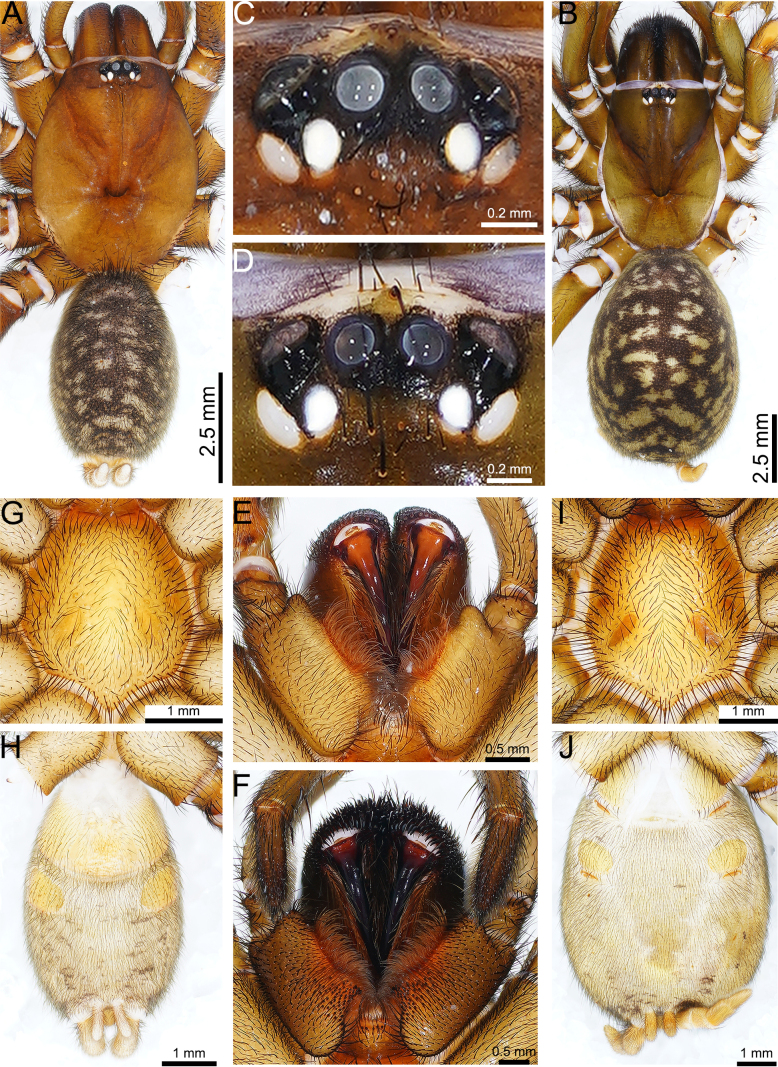
*Damarchus dai* sp. nov. from Jinghong population, male holotype (**A, C, E, G, H**) and female paratype (**B, D, F, I, J**). **A, B**. Habitus; **C, D**. Ocular area; **E, F**. Chelicerae and maxillae; **G, I**. Sternum; **H, J**. Opisthosoma. **A–D**. Dorsal; **E–J**. Ventral.

KYUARA#1497. Coloration. Living spiders (Fig. 1A) carapace and chelicerae dark; palp and legs reddish brown, femora slightly darker than remaining segments; opisthosoma dark brown dorsally with pale spots; paler ventrally, with very sparse irregular brown spots; book lung covers pale yellowish. In ethanol (Fig. 2A, E, G, H), color slightly paler than in life.

Measurements. Jinghong population (*n* = 2): Total length (excluding chelicerae): 9.70 (holotype), 7.75 (paratype KYUARA#1493) Carapace 4.61 ± 0.51 long, 3.62 ± 0.31 wide. Eyes (Fig. 2C). Sizes and interdistances: AME 0.17 ± 0.01, ALE 0.27 ± 0.01, PLE 0.18 ± 0.04, PME 0.16 ± 0.04, AME–AME 0.09 ± 0.01, AME–ALE 0.09 ± 0.04, ALE–PLE 0.07 ± 0.01, PLE–PME 0.04 ± 0.01, PME–AME 0.06 ± 0.01, PME–PME 0.35 ± 0.04; eye group 0.42 ± 0.05 long, wide anteriorly 0.90 ± 0.07, posteriorly 0.87 ± 0.10; MOA 0.38 ± 0.05 long, wide anteriorly 0.42 ± 0.04, posteriorly 0.60 ± 0.05. Labium (Fig. 2E) l.03 ± 0.12 long, 0.40 ± 0.03 wide. Maxillae (Fig. 2E) 1.40 ± 0.27 long, 0.73 ± 0.14 wide. Sternum (Fig. 2G) 2.35 ± 0.35 long, 1.92 ± 0.40 wide. For measurements of palp and legs see Table 1.

**Table 1.** *Damarchus dai* sp. nov., Jinghong population, male palp, and legs measurements (*n* = 2).

**Segment**	**Femur**	**Patella**	**Tibia**	**Metatarsus**	**Tarsus**	**Total**
**Appendage**						
**Palp**	1.70 ± 0.14 (1.60–1.80)	1.00 ± 0.14 (0.90–1.10)	1.64 ± 0.29 (1.43–1.84)		0.70 ± 0.04 (0.67–0.72)	**5.03 ± 0.61 (4.60–5.46)**
**Leg I**	3.73 ± 0.32 (3.50–3.95)	1.97 ± 0.30 (1.75–2.18)	3.00 ± 0.47 (2.67–3.33)	2.81 ± 0.52 (2.44–3.17)	1.95 ± 0.29 (1.74–2.15)	**13.44 ± 1.90 (12.10–14.78)**
**Leg II**	3.47 ± 0.40 (3.18–3.75)	1.73 ± 0.32 (1.50–1.95)	2.75 ± 0.53 (2.37–3.12)	2.69 ± 0.60 (2.26–3.11)	1.88 ± 0.32 (1.65–2.10)	**12.50 ± 2.17 (10.96–14.03)**
**Leg III**	3.03 ± 0.34 (2.79–3.27)	1.59 ± 0.27 (1.40–1.78)	2.10 ± 0.43 (1.79–2.40)	2.98 ± 0.53 (2.60–3.35)	1.87 ± 0.33 (1.64–2.10)	**11.56 ± 1.89 (10.22–12.90)**
**Leg IV**	3.92 ± 0.08 (3.86–3.97)	1.62 ± 0.44 (1.31–1.93)	3.48 ± 0.66 (3.01–3.94)	4.09 ± 0.30 (3.88–4.30)	1.98 ± 0.45 (1.66–2.30)	**15.08 ± 1.92 (13.72–16.44)**

Menglun population (KYUARA#1492, Fig. 7A): Total length (excluding chelicerae) 8.97. Carapace 5.00 long, 3.97 wide. Eyes (Fig. 7C). Sizes and interdistances: AME 0.19, ALE 0.30, PLE 0.21, PME 0.18, AME–AME 0.07, AME–ALE 0.10, ALE–PLE 0.07, PLE–PME 0.05, PME–AME 0.08, PME–PME 0.38; eye group 0.50 long, wide anteriorly 0.95, posteriorly 0.92; MOA 0.48 long, wide anteriorly 0.47, posteriorly 0.65. For measurements of palp and legs see Table 2.

**Table 2.** *Damarchus dai* sp. nov., Menglun population, male palp, and legs measurements (*n* = 1).

**Segment**	**Femur**	**Patella**	**Tibia**	**Metatarsus**	**Tarsus**	**Total**
**Appendage**						
**Palp**	2.25	1.18	1.93		0.83	**6.19**
**Leg I**	4.32	2.30	3.35	3.25	2.36	**15.58**
**Leg II**	4.13	2.08	2.97	3.10	2.12	**14.40**
**Leg III**	3.50	1.75	2.27	3.43	2.14	**13.09**
**Leg IV**	4.93	2.16	4.06	4.83	2.27	**18.25**

Carapace (Fig. 2A) relatively smooth, bearing a narrow membranous region laterally on its outer margin, near coxae III. Setae on carapace generally sparse, bristles relatively distinct along outer margin of carapace, on cephalic region, and on posterior part of thoracic region; those on cephalic region forming a median longitudinal row. Cephalic region not obviously raised. Eyes (Fig. 2A, C) on low mound, posterior eyes noticeably whiter than anterior eyes. Fovea (Fig. 2A) procurved, width ~ 1/2–2/3 width of the ocular area.

Chelicerae. Paturon (Fig. 2E) with seven or eight promarginal teeth in one row (the eighth tooth smallest), the remaining teeth relatively similar in size; mesobasal denticles present adjacent to the first-third proximal teeth, numbering more than 20; dorsally bearing a band of bristles that becomes broader and denser from the proximal to the distal end; distal bristles not forming a typical rastellum. Maxillae (Fig. 2E) possessing distinct anterior lobe and posterior heel. Cuspules on maxillae and labium peg-like, notably smaller, more tenuous, and less distinct compared to those of females; the number of cuspules on maxillae shows significant variation among individuals: KYUARA#1497 50–55; KYUARA#1492 42–45; KYUARA#1493 22–25; labium bearing only two or three cuspules. Three pairs of sternal sigilla (Fig. 2G), become closer to outer margin of sternum from posterior to anterior; posterior pair distinctly larger than anterior two pairs; anterior two pairs nearly equal in size; anteriormost pair almost merging into outer margin of sternum. All sternal sigilla less sclerotized and with less defined margins compared to those of females (Fig. 2I). Opisthosoma (Fig. 2H) oval. Four spinnerets (*n* = 3): PMS: 0.59 ± 0.15 long, 0.19 ± 0.03 diameter, PLS: 1.78 ± 0.45 long, 0.37 ± 0.13 maximal diameter, length of basal, medial, and apical segments 0.87 ± 0.24, 0.48 ± 0.14, 0.42 ± 0.07, respectively. Apical segment digitiform.

**Figure 3.**
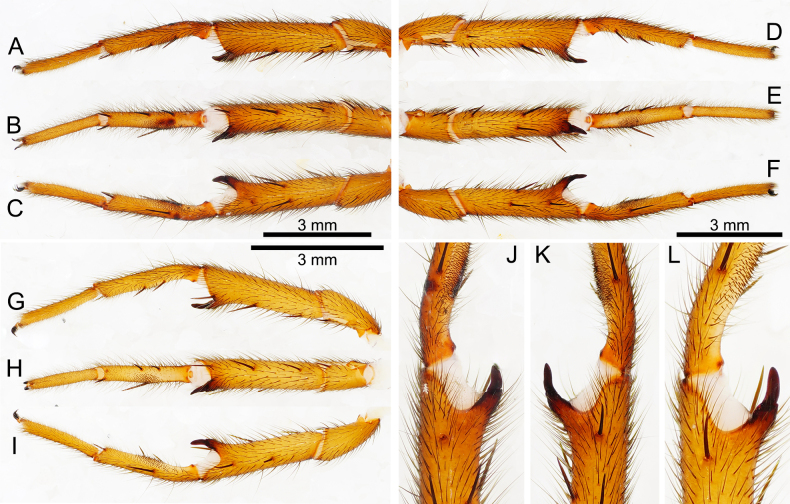
*Damarchus dai* sp. nov. from Jinghong population, male secondary sexual characteristics on legs I. **A–F, J, K**. Male holotype; **G–I, L**. Male paratype; **A–C**. Left side of holotype; **D–F**. Right side of holotype; **G–I**. Left side of paratype; **J**. Holotype left tibial spur; **K**. Holotype right tibial spur; **L**. Paratype left tibial spur; **C, F, I, J–L**. Prolateral; **B, E, H**. Ventral; **A, D, G**. Retrolateral.

Spination of anterior legs (including large spine-like bristles; Figs 3, 8). General: Leg I, femur pd (0–1–1); patella pd (0–0–1), rv (0–0–1); tibia pl (–1–1–), pv (0–0–Sp), rv (1–1–1), rl (1–0–0); metatarsus pl (0–1–0), pv (0–0–1), rv (1–1–1). Leg II, femur pd (1–1–1); patella pd (0–0–1), rv (0–0–1); tibia pl (–1–1–), pv (0–0–2), rv (1–1–1), rl (1–0–0); metatarsus pl (0–1–0), pv (0–0–1), rv (1–1–2). Variations: holotype, rv as (0–1–1) on right patella I, pv as (0–1–Sp) on both tibiae I, pd as (0–1–1) on left patella II, pv as (0–1–1) on left tibia II; paratype, with additional ds (–1–1–) on left femur I, without pd on left patella I, without rl on left tibia I, with additional ds (–1–1–), rd (–1–1–) on left femur II, without pd on left patella II, pv as (0–0–1) and rv as (2–1–1) on left tibia II, rv as (1–1–1) on left metatarsus II; non-type (KYUARA#1492, Fig. 8), with additional ds (1–1–1) on both femora I, with additional ds (1–1–1) on both femora II, rv as (1–1–1) on right metatarsus II.

Leg scopulae. On tarsi I–IV and distal half of metatarsi I and II.

Trichobothria. Generally, 16–20 in two rows on tibiae, fewer than ten in one row on metatarsi, and in one zigzag row on tarsi, respectively.

Metatarsal preening combs. On legs II–IV; leg II with Pv comb only, close to base of Pv spine (weakly developed, resembling bristles or inconspicuous); legs III–IV with both Pv and Rv combs, close to base of Pv and Rv spines, respectively; each comb commonly comprised of 3 teeth (short spines), except for 4 teeth on all combs on metatarsi III of KYUARA#1492.

Claws with ~ 9 teeth in one S-shaped row on all paired claws, proximal tooth smallest. Unpaired claw present on all tarsi and edentate.

Secondary sexual characteristics (Figs 3J–K, 8G–H). Coupling spur on distal part of tibia I, with one stout spine (2–3 × longer than wide) distally. Sub-proximal portion of metatarsus I curved in lateral view; patch of short stout setae developing on proximal one-third to half portion of Pv side of metatarsus I.

Palp (Figs 5, 10A–C). Tibia with two strong bristles near Pv side, lacks a retrolateral bump on Rl side. Tegulum bulbous, large, and wide; embolus long, with length ~ 1.5 × the width of the tegulum; no laminae or ribs on lower side, no para-embolic apophysis.

**Figure 4.**
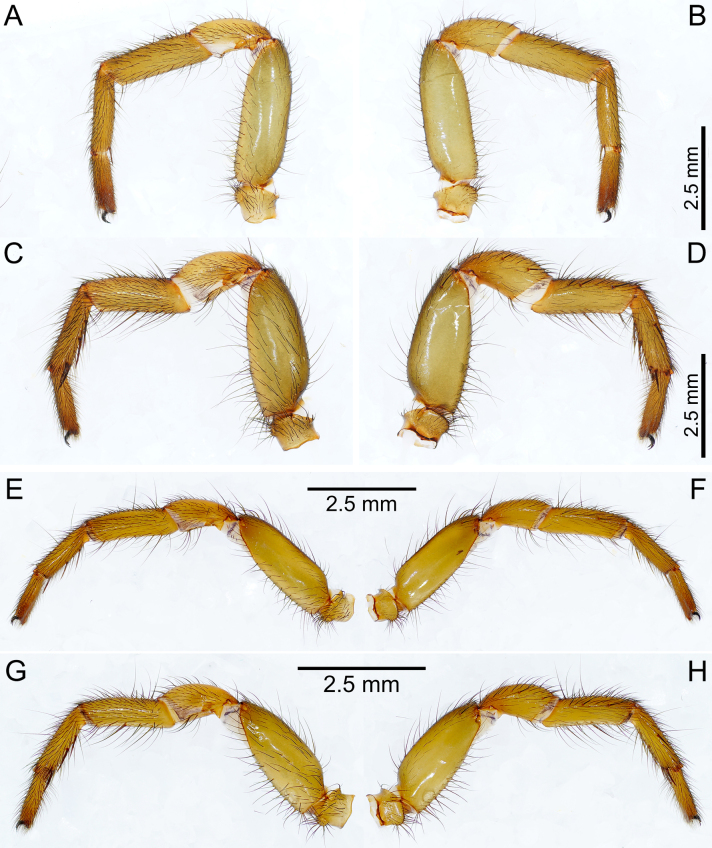
*Damarchus dai* sp. nov. from Jinghong population, female legs of KYUARA#1498 (**A–D**) and KYUARA#1494 (**E–H**). **A, B, E–F**. Legs I; **C, D, G–H**. Legs II; **A, C, E, G**. Retrolateral; **B, D, F, H**. Prolateral.

**Figure 5.**
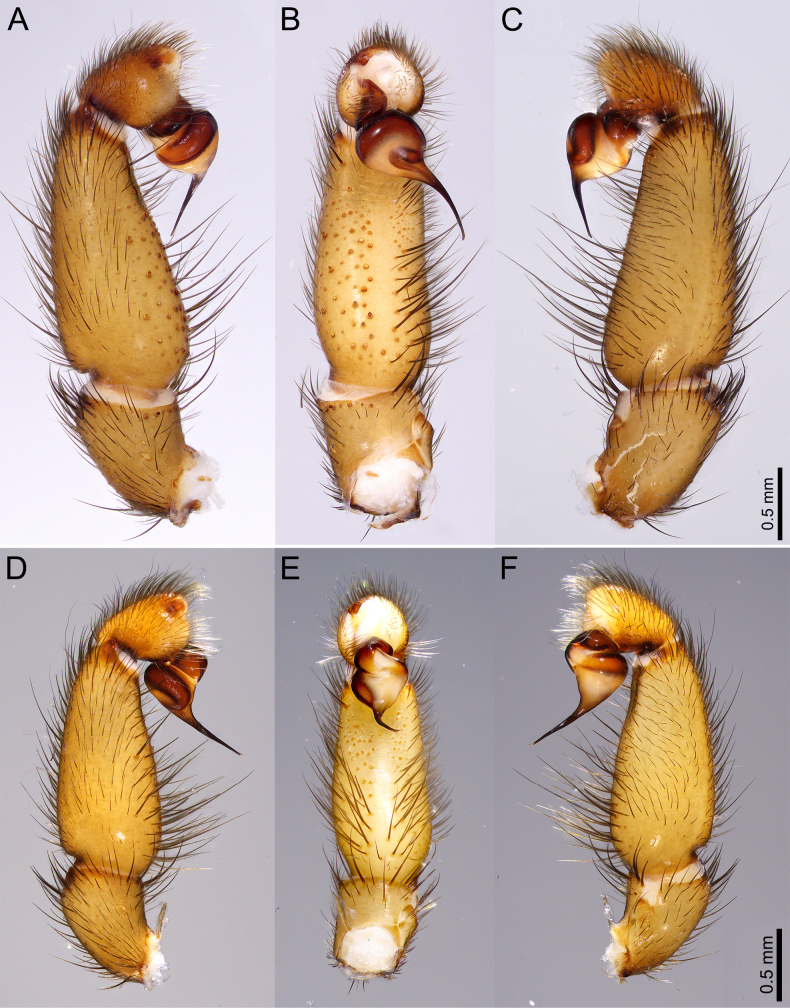
*Damarchus dai* sp. nov. from Jinghong population, male palps of holotype (**A–C**) and paratype (**D–F**). **A, D**. Prolateral; **B, E**. Ventral; **C, F**. Retrolateral.

##### Description of female.

KYUARA#1498. Coloration. Living spiders (Fig. 1B) carapace and chelicerae dark; palp and legs light brown, femora slightly darker than remaining segments; opisthosoma dark brown dorsally, with pale spots; paler ventrally, with almost no brown spots; book lung covers pale yellowish. In ethanol (Fig. 2B, F, I, J), color slightly paler than in life.

Measurements. Jinghong population (*n* = 2): Total length (excluding chelicerae): 12.50 (paratype KYUARA#1498), 9.09 (paratype KYUARA#1494) Carapace 4.92 ± 0.83 long, 3.80 ± 0.61 wide. Eyes (Fig. 2D). Sizes and interdistances: AME 0.16 ± 0.01, ALE 0.27 ± 0.02, PLE 0.28 ± 0.02, PME 0.16 ± 0.01, AME–AME 0.11 ± 0.01, AME–ALE 0.11 ± 0.04, ALE–PLE 0.08 ± 0.03, PLE–PME 0.03 ± 0.00, PME–AME 0.09 ± 0.06, PME–PME 0.41 ± 0.07; eye group 0.49 ± 0.11 long, wide anteriorly 1.02 ± 0.16, posteriorly 1.02 ± 0.22; MOA 0.46 ± 0.09 long, wide anteriorly 0.45 ± 0.08, posteriorly 0.67 ± 0.12. Labium (Fig. 2F) 1.08 ± 0.25 long, 0.57 ± 0.16 wide. Maxillae (Fig. 2F) 1.89 ± 0.45 long, 1.03 ± 0.18 wide. Sternum (Fig. 2I) 2.80 ± 0.57 long, 2.19 ± 0.13 wide. For measurements of palp and legs see Table 3.

**Table 3.** *Damarchus dai* sp. nov., Jinghong population, female palp, and legs measurements (*n* = 2).

**Segment**	**Femur**	**Patella**	**Tibia**	**Metatarsus**	**Tarsus**	**Total**
**Appendage**						
**Palp**	2.24 ± 0.48 (1.90–2.58)	1.33 ± 0.29 (1.12–1.53)	1.44 ± 0.28 (1.24–1.63)		1.35 ± 0.22 (1.19–1.50)	**6.35 ± 1.27 (5.45–7.24)**
**Leg I**	3.13 ± 0.53 (2.75–3.50)	1.63 ± 0.39 (1.35–1.90)	2.00 ± 0.35 (1.75–2.25)	1.69 ± 0.19 (1.55–1.82)	1.23 ± 0.18 (1.10–1.35)	**9.66 ± 1.64 (8.50–10.82)**
**Leg II**	2.85 ± 0.58 (2.44–3.26)	1.89 ± 0.58 (1.48–2.30)	1.90 ± 0.42 (1.60–2.20)	1.75 ± 0.49 (1.40–2.10)	1.29 ± 0.21 (1.14–1.43)	**9.68 ± 2.28 (8.06–11.29)**
**Leg III**	2.59 ± 0.76 (2.05–3.12)	1.56 ± 0.56 (1.16–1.95)	1.26 ± 0.15 (1.15–1.36)	1.95 ± 0.28 (1.75–2.15)	1.33 ± 0.18 (1.20–1.46)	**8.68 ± 1.93 (7.31–10.04)**
**Leg IV**	3.53 ± 0.88 (2.90–4.15)	2.00 ± 0.62 (1.56–2.43)	2.60 ± 0.47 (2.27–2.93)	2.88 ± 0.54 (2.50–3.26)	1.55 ± 0.38 (1.28–1.82)	**12.55 ± 2.89 (10.51–14.59)**

Menglun population (KYUARA#1524, this specimen died due to dehydration, after that immersed in ethanol, Fig. 7B). Total length (excluding chelicerae) 12.69. Carapace 6.97 long, 6.57 wide. Eyes (Fig. 7D). Sizes and interdistances: AME 0.23, ALE 0.33, PLE 0.35, PME 0.26, AME–AME 0.13, AME–ALE 0.15, ALE–PLE 0.08, PLE–PME 0.03, PME–AME 0.09, PME–PME 0.52; eye group 0.67 long, wide anteriorly 1.34, posteriorly 1.36; MOA 0.62 long, wide anteriorly 0.63, posteriorly 0.85. For measurements of palp and legs see Table 4.

**Table 4.** *Damarchus dai* sp. nov., Menglun population, female palp, and legs measurements (*n* = 1).

**Segment**	**Femur**	**Patella**	**Tibia**	**Metatarsus**	**Tarsus**	**Total**
**Appendage**						
**Palp**	3.47	1.90	2.32		1.92	**9.61**
**Leg I**	5.12	3.21	3.30	2.86	1.86	**16.35**
**Leg II**	4.65	2.92	2.80	2.60	1.80	**14.77**
**Leg III**	3.62	2.60	1.80	2.88	1.83	**12.73**
**Leg IV**	5.40	3.17	3.72	4.30	1.92	**18.51**

Carapace (Fig. 2B) relatively smooth, bearing narrow, membranous region laterally on its outer margin, near coxae III. Almost no setae on carapace, setae generally sparse along outer margin of carapace, bristles on cephalic region; those on cephalic region forming one median longitudinal row. Cephalic region obviously raised. Eyes (Fig. 2B, D) on low mound, posterior eyes noticeably whiter than anterior eyes. Fovea (Fig. 2B) procurved, width ~ half the width of the ocular area. Chelicerae. Paturon (Fig. 2F) with seven or eight promarginal teeth in one row (the eighth tooth smallest), the remaining teeth relatively similar in size; mesobasal denticles present adjacent to the first-third proximal teeth, numbering more than 20; dorsally bearing a band of bristles that becomes broader and denser from proximal to distal end; distal bristles not forming a typical rastellum. Maxillae (Fig. 2F) possessing distinct anterior lobe and posterior heel. Cuspules on maxillae and labium peg-like, notably bigger, more distinct, stouter, and more strongly sclerotized compared to those of males; the number of cuspules on maxillae shows significant variation among individuals (especially specimen KYUARA#1524): KYUARA#1498 60–65; KYUARA#1494 41–44; KYUARA#1524 123–125; labium bearing 3–6 cuspules. Three pairs of sternal sigilla (Fig. 2I), become closer to outer margin of sternum from posterior to anterior; posterior pair distinctly larger than anterior two pairs; anterior two pairs nearly equal in size; anteriormost pair almost merging into outer margin of sternum. All sternal sigilla more sclerotized and with more defined margins compared to those of males (Fig. 2G). Opisthosoma (Fig. 2J) oval. Four spinnerets (*n* = 3): PMS: 0.76 ± 0.16 long, 0.28 ± 0.06 diameter, PLS: 1.93 ± 0.51 long, 0.52 ± 0.10 maximal diameter, length of basal, medial, and apical segments 0.97 ± 0.28, 0.48 ± 0.16, 0.49 ± 0.12, respectively. Apical segment digitiform.

Spination of anterior legs (including large spine-like bristles; Figs 4, 9). General: Leg I, femur pd (0–0–1); tibia pl (–1–1–), rv (0–1–0); metatarsus pv (0–0–1), rv (1–1–1). Leg II, femur pd (0–0–1); patella pd (–1–1–); tibia pl (1–1–1), rv (0–1–0); metatarsus pl (0–1–0), pv (0–0–2), rv (1–1–2). Variations: paratype (KYUARA#1498), without pl on both tibiae I, pv as (0–0–1) on both metatarsi II; paratype (KYUARA#1494), pl as (1–1–1) on both tibiae I; non-type (KYUARA#1524, Fig. 9), with additional pd (0–1–1) on both patellae I, rv as (1–1–0) on both tibiae I, pd as (0–0–1) on both patellae II, rv as (1–1–1) on both tibiae II, without pl; rv as (1–1–2–2) on both metatarsi II.

Leg scopulae. On tarsi I–IV and distal half of metatarsi I and II.

Trichobothria. Paratype: 16–20 in two rows on tibiae, 8–10 in one row on metatarsi and in one zigzag row on tarsi. Variations: non-type, 18 on I–III metatarsi, 24 on IV metatarsus, 12–18 on I–IV tarsi.

Metatarsal preening combs. On legs II–IV; II with Pv comb only, close to base of Pv spine; III–IV with both Pv and Rv combs, close to base of Pv and Rv spines, respectively; each comb commonly composed of three teeth (short spines). Variations: two teeth on all combs on metatarsi II of KYUARA#1524, 4 teeth on all combs on metatarsi III of KYUARA#1494 and KYUARA#1524.

Claws with two rows of teeth, ~ 5 teeth per row on outer claws and four teeth per row on inner claws on all paired claws, proximal tooth smallest. Unpaired claw present on all tarsi and edentate.

**Figure 6.**
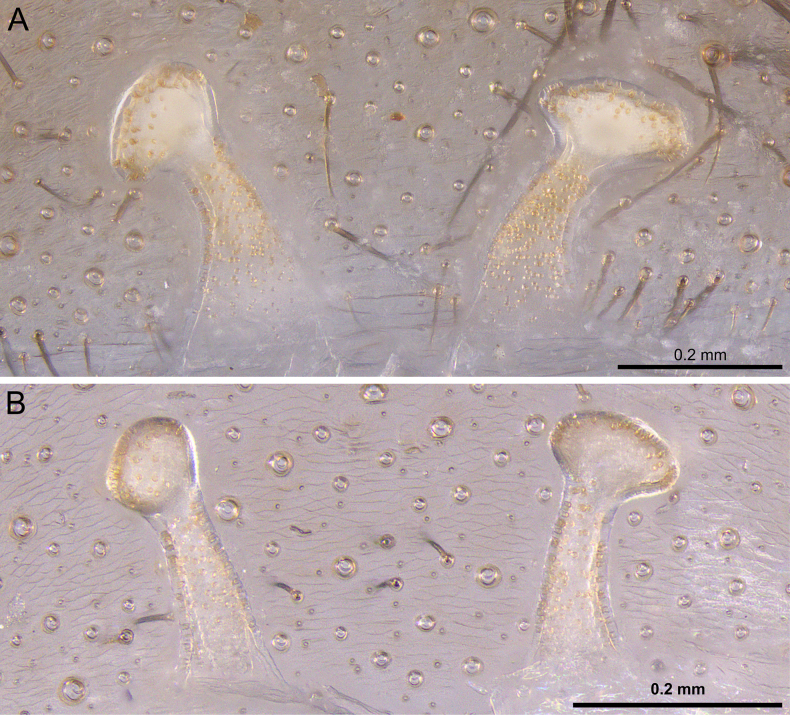
*Damarchus dai* sp. nov. from Jinghong population. Spermathecae of paratypes. **A**. KYUARA#1498; **B**. KYUARA#1494.

Spermathecae (Fig. 6). More or less clavate-shaped. Stalk densely covered with glandular pores, broad at base, distinctly constricted toward upper 2/3; the receptacle, bearing glandular pores only on the uppermost surface, with lower 2/3 of region devoid of pores. Variations: non-type (KYUARA#1524, Fig. 10D), the number and density of glandular pores on the stalk, as well as the degree of sclerotization, are greater than those observed in paratypes, the receptacle is crater-shaped, with a centrally depressed area and a raised margin; the marginal area is sclerotized and bears glandular pores.

**Figure 7.**
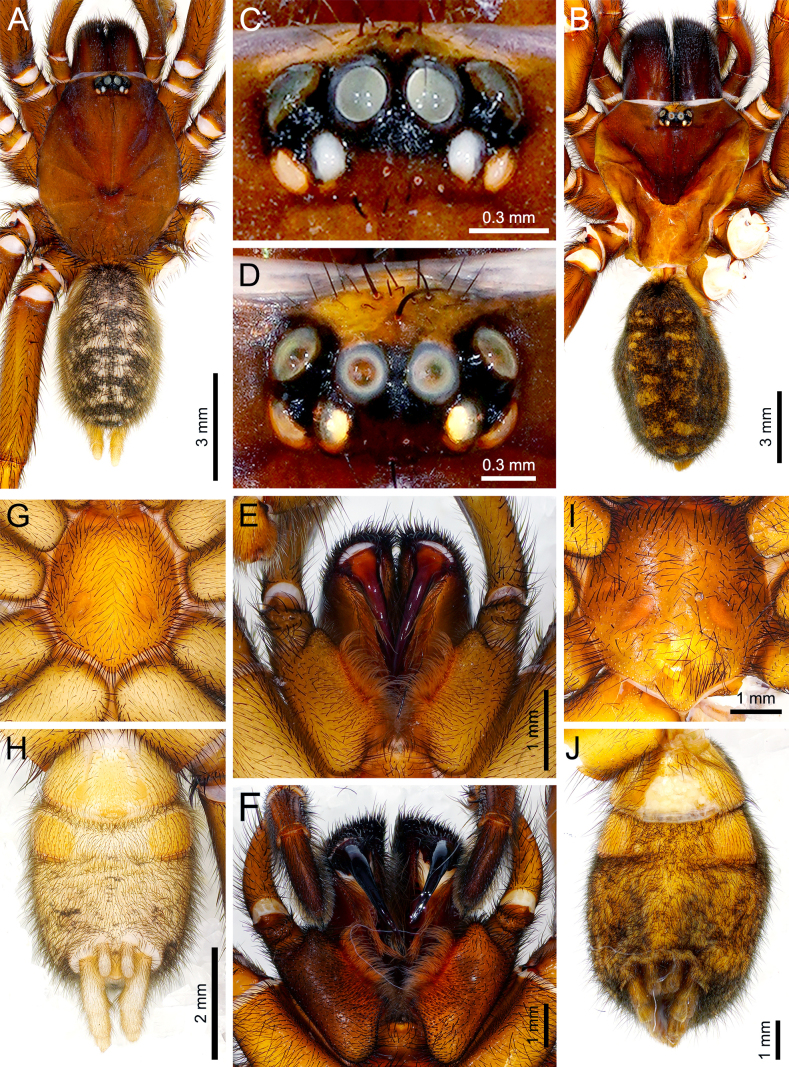
*Damarchus dai* sp. nov. from Menglun population, male (KYUARA#1492, **A, C, E, G–H**) and female (KYUARA#1524, **B, D, F, I–J**). **A, B**. Habitus; **C, D**. Ocular area; **E, F**. Chelicerae and maxillae; **G, I**. Sternum; **H, J**. Opisthosoma. **A–D**. Dorsal; **E–J**. Ventral.

**Figure 8.**
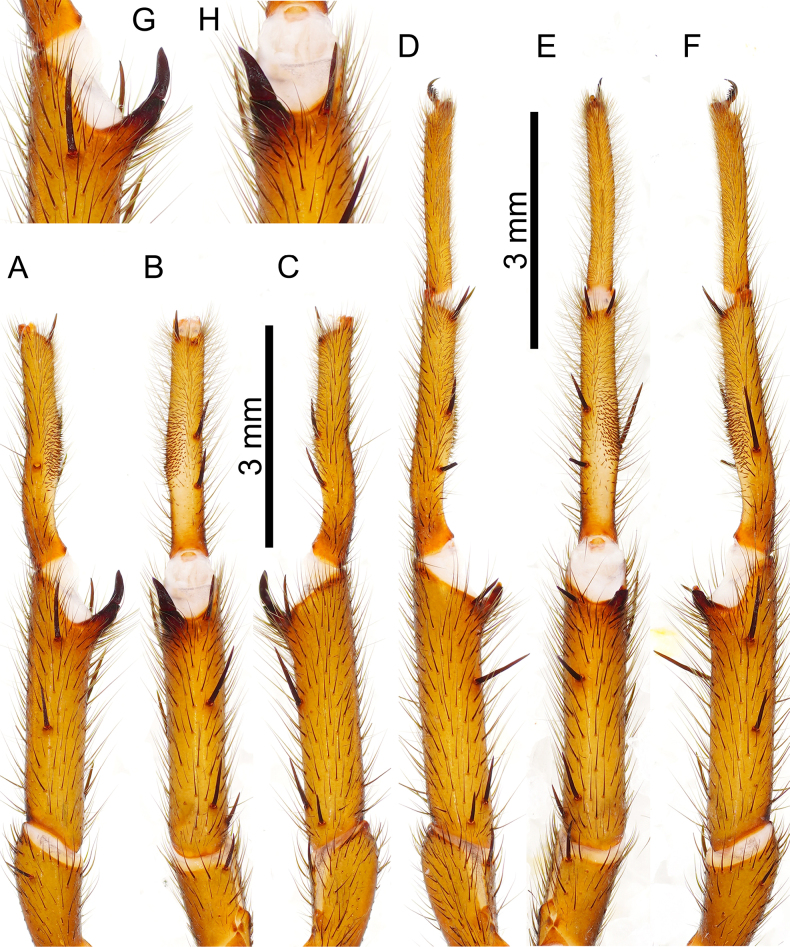
*Damarchus dai* sp. nov. from Menglun population, male (KYUARA#1492) secondary sexual characteristics on legs I. **A–C, G–H**. Left side; **D–F**. Right side; **A, F, G**. Prolateral; **B, E, H**. Ventral; **C, D**. Retrolateral.

**Figure 9.**
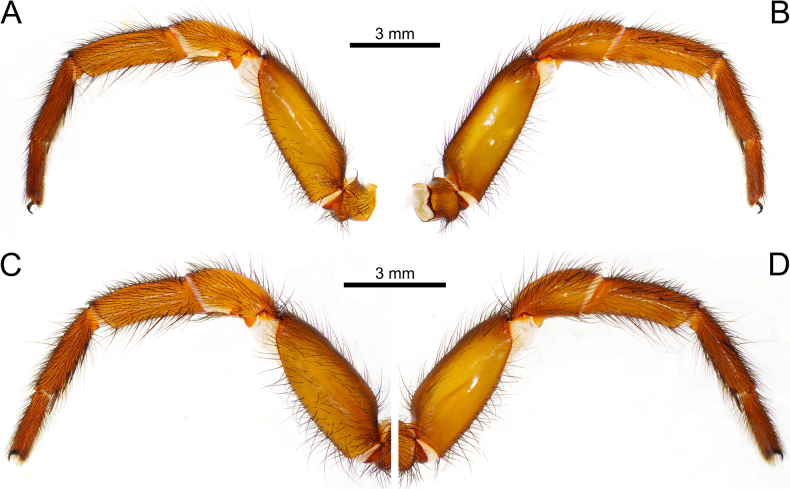
*Damarchus dai* sp. nov. from Menglun population, female (KYUARA#1524) legs. **A, B**. Legs I; **C, D**. Legs II; **A, C**. Retrolateral; **B, D**. Prolateral.

**Figure 10.**
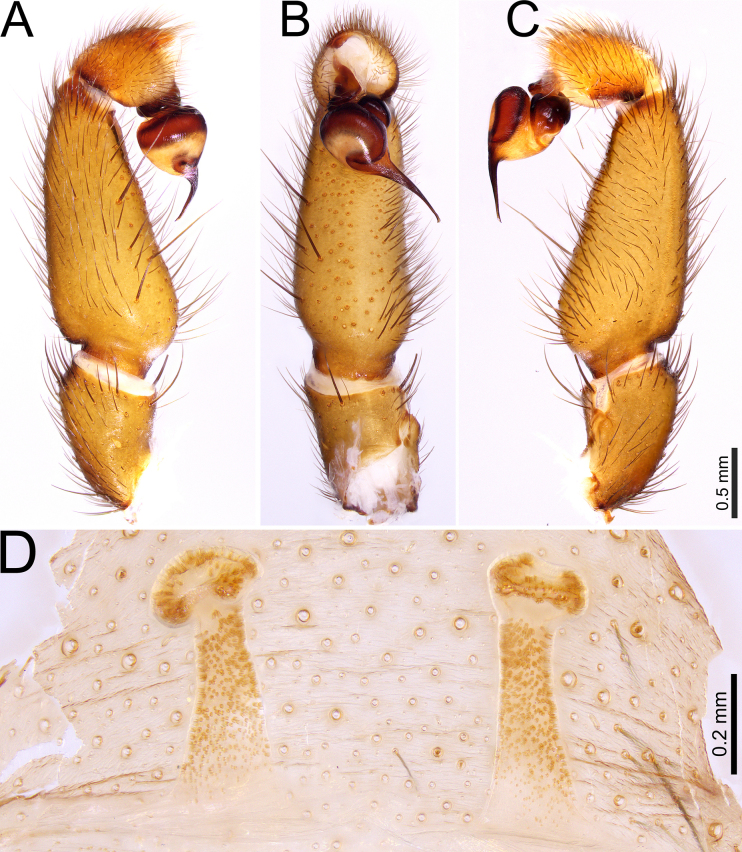
*Damarchus dai* sp. nov. from Menglun population, male (KYUARA#1492) palps (**A–C**) and female (KYUARA#1524) spermathecae (**D**). **A**. Prolateral; **B**. Ventral; **C**. Retrolateral.

##### Etymology.

The specific epithet refers to the Dai people, the main ethnic group inhabiting Xishuangbanna Dai Autonomous Prefecture, the type locality of this new species. Noun in apposition.

##### Remarks.

All examined specimens were collected from Xishuangbanna and are here considered to represent two geographical populations: the Jinghong population (type series, 2 ♂ 2 ♀ examined) and the Menglun population (non-type specimens, 1 ♂ 1 ♀ examined). The *COI* genetic distance (Table 13) within the Jinghong population ranges from 0.3–5.2% (mean distances 3.45%), while that within the Menglun population is 1.9%. A relatively significant difference is observed between the Jinghong and Menglun populations, with a genetic distance reaching from 10.6–12.2% (mean distances ~ 11.2%), which exceeds the interspecific thresholds (i.e., 2–6% or 9.5%) used in some previous mygalomorph studies (Hamilton et al. 2011; Castalanelli et al. 2014; Ortiz and Francke 2016). However, no reliable morphological differences were identified between these two populations based on the available specimens, particularly in the key sexual features (i.e., female vulva, male palpal organ and coupling spur). The female from Menglun (KYUARA#1524) exhibits stronger sclerotization at the distal part of the receptacle of spermathecae and closer proximity of the third pair of sigilla on sternum compared to the two females from Jinghong (Figs 2I, 7I); however, these differences cannot be ruled out as potentially related to individual age and developmental stage, especially given that KYUARA#1524 is the largest female (according to total length and carapace size) among them. Due to their relatively conservative life history, mygalomorphs often exhibit high levels of genetic structuring among populations, with intraspecific *COI* genetic distances among different populations often comparable to interspecific distances (Satler et al. 2011).

Given the lack of identifiable morphological distinctions, we consider it more appropriate to treat the Jinghong and Menglun populations as the same species in the current taxonomy of *Damarchus*, rather than splitting them based solely on the currently available molecular data. We also acknowledge that with the acquisition of more specimens in future studies, *D. erebus* may prove to contain more than a single species.

#### Damarchus erebus sp. nov.

Taxon classificationAnimaliaAraneaeBemmeridae

0FE48AEF-31FB-5245-8552-FAF578D26FF6

https://zoobank.org/80A6CC12-4025-49BE-A94E-2E3DD5484DF3

Figs 11, 12, 13, 14, 15, 16, 17, 18, 19, 20, 21, Tables 5–8

##### Chinese name.

暗达玛蛛.

##### Type material.

***Holotype***: China • ♂; Yunnan Province, Dehong Dai and Jingpo Autonomous Prefecture, Lianghe County, Kazi Village; 24.601667°N, 98.239444°E, alt. 1412 m; 13 Apr. 2023, W. Wang and Z. Yang leg.; GenBank: PX967108; KYUARA#1509. ***Paratypes***: China • 1 ♂; same data as preceding, but Sanjia Village; 24.7504°N, 98.2102°E; 5 Feb. 2023, Y. Lin leg.; GenBank: PX967106; KYUARA#1495. • 1 ♂; same data as preceding, but near Zhaojia Village; 24.7503°N, 98.2101°E, alt. 1732 m; 24 Mar. 2023, Y. Lin leg.; GenBank: PX967107; KYUARA#1496. • 1 ♂; same data as holotype, but Apr. 2021, alt. 1580 m, Y. Lin leg.; KYUARA#1504. • 2 ♀♀; same data as KYUARA#1504; KYUARA#1505–1506. • 3 ♀♀; same data as the holotype; GenBank: PX967109 (KYUARA#1514); KYUARA#1513–1515.

##### Other material examined.

China • 1 ♂; Yunnan Province, Dehong Dai and Jingpo Autonomous Prefecture, Yingjiang County, TPNR, 3 May 2023, Z. Li leg.; GenBank: PX967110; KYUARA#1531. • 2 ♂♂; same data as locality, without date; KYUARA#1539; GenBank: PX967102, KYUARA1540. • 1 ♀; same data as preceding but along Provincial Road 318 (in TPNR); 16 Apr. 2023; 24.628817°N, 97.767325°E, alt. 1408 m; W. Wang & Z. Yang leg.; GenBank: PX967103; KYUARA#1526.

##### Diagnosis.

*D. erebus* sp. nov. shares a similar overall appearance with *D. dai* sp. nov., particularly in the coloration of the female legs, where the femora are distinctly darker than the remaining segments. Moreover, *D. erebus* sp. nov. can be distinguished from *D. dai* by: (1) the short embolus, with length ~ equal to the width of the tegulum (vs ~ 1.5 × in *D. dai*); (2) the presence of a retrolateral bump on male palpal tibia (vs lack in *D. dai*); (3) the short, distinctly everted spermathecal stalk, similar in length to the receptacle (vs the stalk is long, ~ 2–3 × the length of the receptacle in *D. dai*).

##### Description of male.

**Figure 11.**
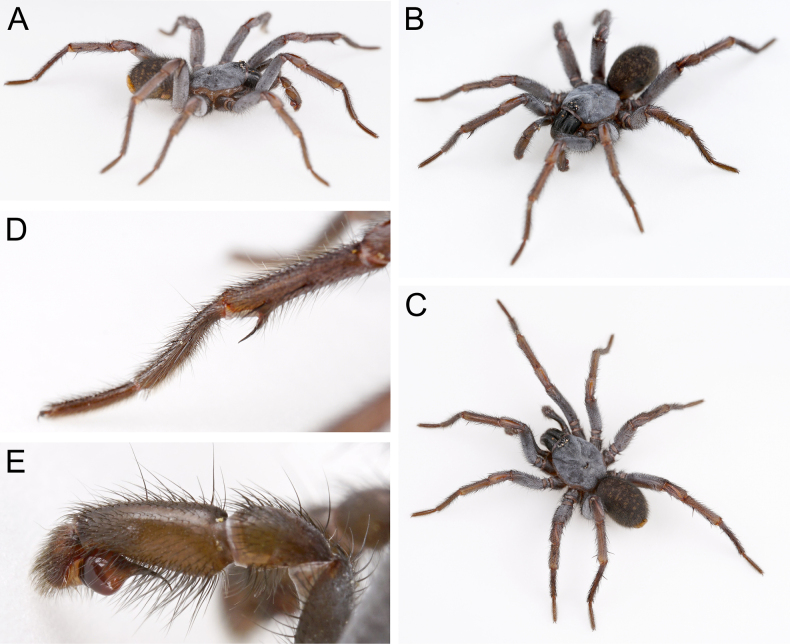
*Damarchus erebus* sp. nov., living spiders from Lianghe population, male holotype (KYUARA#1509, **A–C**). **A**. Lateral; **B**. Anterodorsal; **C**. Dorsal; **D**. Coupling spur, prolateral; **E**. Palp, retrolateral.

**Figure 12.**
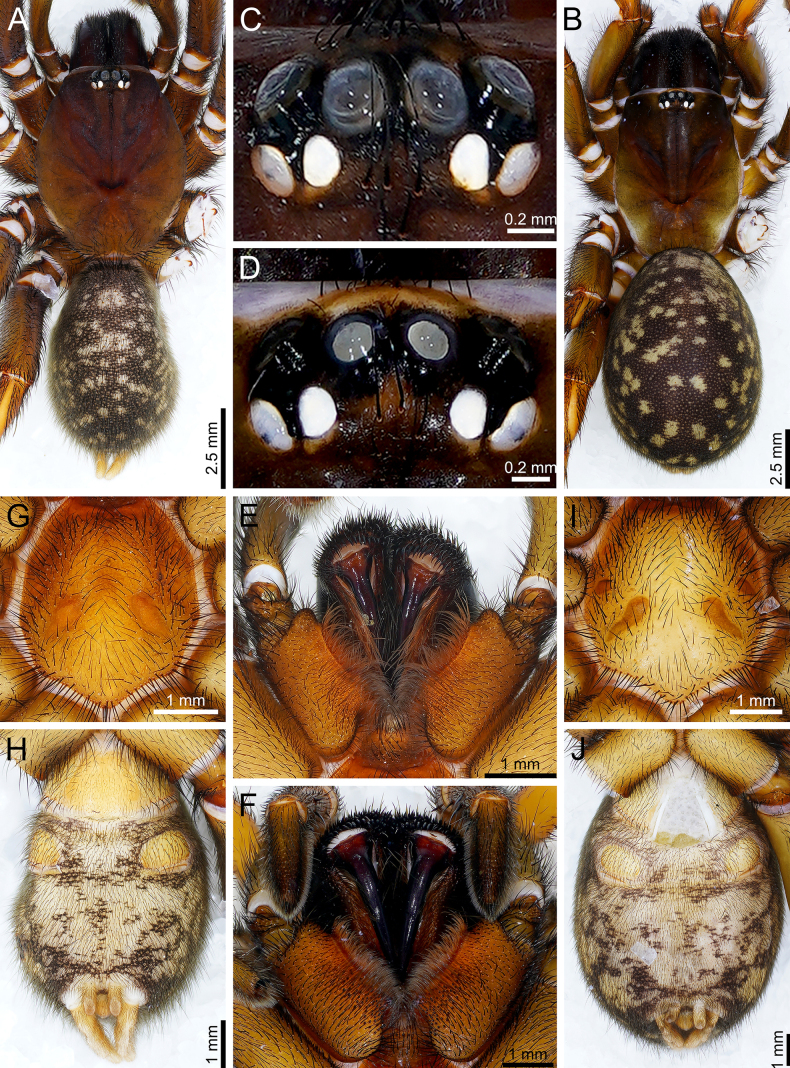
*Damarchus erebus* sp. nov. from Lianghe population, male holotype (**A, C, E, G–H**) and female paratype (**B, D, F, I–J**). **A, B**. Habitus; **C, D**. Ocular area; **E, F**. Chelicerae and maxillae; **G, I**. Sternum; **H, J**. Opisthosoma; **A–D**. Dorsal; **E–J**. Ventral.

KYUARA#1509. Coloration. Living spiders (Fig. 11) carapace and chelicerae dark; carapace and femora covered with whitish frosted coating; palp and legs brown, femora slightly darker than remaining segments; opisthosoma dark brown dorsally, with pale spots; paler ventrally, with some irregular brown spots; book lung covers pale yellowish. In ethanol (Fig. 12A, E, G, H), color slightly paler than in life; whitish frosted coating not obvious; overall coloration of the Yingjiang population is darker than that of the Lianghe population.

Measurements. Lianghe population (*n* = 4): Total length (excluding chelicerae): 10.61 ± 1.03 (9.50–11.61) (including holotype, paratypes: KYUARA#1495–1496 and KYUARA#1504). Carapace 5.67 ± 0.30 long, 4.64 ± 0.22 wide. Eyes (Fig. 12C). Sizes and interdistances: AME 0.22 ± 0.02, ALE 0.33 ± 0.01, PLE 0.24 ± 0.02, PME 0.21 ± 0.01, AME–AME 0.13 ± 0.03, AME–ALE 0.11 ± 0.03, ALE–PLE 0.10 ± 0.02, PLE–PME 0.05 ± 0.02, PME–AME 0.10 ± 0.03, PME–PME 0.47 ± 0.02; eye group 0.58 ± 0.01 long, wide anteriorly 1.15 ± 0.02, posteriorly 1.18 ± 0.02; MOA 0.54 ± 0.03 long, wide anteriorly 0.55 ± 0.05, posteriorly 0.78 ± 0.03. Labium (Fig. 12E) 1.14 ± 0.06 long, 0.48 ± 0.02 wide. Maxillae (Fig. 12E) 2.01 ± 0.05 long, 0.95 ± 0.06 wide. Sternum (Fig. 12G) 3.00 ± 0.12 long, 2.69 ± 0.11 wide. For measurements of palp and legs see Table 5.

**Table 5.** *Damarchus erebus* sp. nov., Lianghe population, male palp, and legs measurements (*n* = 4).

**Segment**	**Femur**	**Patella**	**Tibia**	**Metatarsus**	**Tarsus**	**Total**
**Appendage**						
**Palp**	2.61 ± 0.13 (2.47–2.75)	1.34 ± 0.10 (1.20–1.43)	2.32 ± 0.06 (2.27–2.40)		0.85 ± 0.04 (0.80–0.88)	**7.12 ± 0.26 (6.85–7.44)**
**Leg I**	5.09 ± 0.13 (4.95–5.24)	2.56 ± 0.19 (2.30–2.75)	4.05 ± 0.10 (4.00–4.20)	3.58 ± 0.06 (3.50–3.65)	2.46 ± 0.06 (2.40–2.54)	**17.74 ± 0.45 (17.29–18.38)**
**Leg II**	4.58 ± 0.09 (4.50–4.70)	2.33 ± 0.19 (2.05–2.50)	3.36 ± 0.08 (3.26–3.47)	3.27 ± 0.03 (3.23–3.30)	2.30 ± 0.07 (2.23–2.40)	**15.85 ± 0.36 (15.50–16.37)**
**Leg III**	3.89 ± 0.09 (3.78–4.00)	1.99 ± 0.13 (1.80–2.13)	2.52 ± 0.13 (2.35–2.67)	3.46 ± 0.11 (3.30–3.57)	2.29 ± 0.03 (2.25–2.32)	**14.15 ± 0.41 (13.58–14.55)**
**Leg IV**	5.33 ± 0.24 (5.08–5.60)	2.41 ± 0.10 (2.30–2.50)	4.18 ± 0.09 (4.10–4.28)	5.20 ± 0.17 (5.00–5.40)	2.49 ± 0.11 (2.36–2.63)	**19.61 ± 0.29 (19.30–19.93)**

Yingjiang population (*n* = 3): Total length (excluding chelicerae): 10.07 ± 1.04 (9.03–11.10) (KYUARA#1531, is conspicuously smaller than KYUARA#1539–1540, a difference readily apparent by direct comparison). Carapace 5.90 ± 0.70 long, 4.63 ± 0.60 wide. Eyes (Fig. 17C). Sizes and interdistances: AME 0.26 ± 0.03, ALE 0.33 ± 0.03, PLE 0.24 ± 0.04, PME 0.23 ± 0.03, AME–AME 0.12 ± 0.04, AME–ALE 0.09 ± 0.03, ALE–PLE 0.09 ± 0.04, PLE–PME 0.04 ± 0.01, PME–AME 0.09 ± 0.03, PME–PME 0.44 ± 0.10; eye group 0.57 ± 0.07 long, wide anteriorly 1.16 ± 0.13, posteriorly 1.16 ± 0.15; MOA 0.54 ± 0.07 long, wide anteriorly 0.59 ± 0.04, posteriorly 0.80 ± 0.07. For measurements of palp and legs see Table 6.

**Table 6.** *Damarchus erebus* sp. nov., Yingjiang population, male palp, and legs measurements (*n* = 3).

**Segment**	**Femur**	**Patella**	**Tibia**	**Metatarsus**	**Tarsus**	**Total**
**Appendage**						
**Palp**	2.55 ± 0.25 (2.24–2.74)	1.35 ± 0.27 (1.13–1.67)	1.97 ± 0.31 (1.67–2.29)		0.96 ± 0.13 (0.80–1.05)	**6.83 ± 0.85 (5.96–7.65)**
**Leg I**	4.87 ± 0.58 (4.23–5.39)	2.39 ± 0.34 (2.16–2.82)	3.66 ± 0.49 (3.15–4.12)	3.35 ± 0.50 (2.76–3.75)	2.30 ± 0.31 (2.00–2.62)	**16.57 ± 2.23 (14.30–18.70)**
**Leg II**	4.39 ± 0.59 (3.73–4.89)	2.20 ± 0.40 (1.90–2.67)	3.15 ± 0.48 (2.64–3.60)	3.19 ± 0.54 (2.60–3.68)	2.26 ± 0.31 (1.95–2.57)	**15.18 ± 2.26 (12.95–17.41)**
**Leg III**	3.59 ± 0.44 (3.20–4.08)	1.82 ± 0.36 (1.60–2.26)	2.23 ± 0.39 (1.78–2.56)	3.32 ± 0.55 (2.66–3.74)	2.07 ± 0.30 (1.86–2.46)	**13.04 ± 2.03 (11.10–15.10)**
**Leg IV**	5.08 ± 0.74 (4.35–5.78)	2.24 ± 0.42 (1.93–2.78)	3.92 ± 0.59 (3.39–4.57)	5.07 ± 0.86 (4.10–5.74)	2.35 ± 0.36 (2.00–2.70)	**18.65 ± 2.90 (15.84–21.57)**

Carapace (Fig. 12A) relatively smooth, bearing a narrow membranous region laterally along the outer margin, near coxae III. Setae on carapace generally sparse, bristles relatively distinct along outer margin of carapace, on cephalic region, and on posterior part of thoracic region; those on cephalic region forming a median longitudinal row. Cephalic region not obviously raised. Eyes (Fig. 12A, C) on low mound, posterior eyes noticeably whiter than anterior eyes. Variations: Yingjiang population (Fig. 17A, C) posterior eyes noticeably whiter than anterior eyes, AME whiter than ALE. Fovea (Fig. 12A) procurved, width ~ 1/2–2/3 width of the ocular area. Chelicerae. Paturon (Fig. 12E) with eight or nine promarginal teeth in one row (the ninth tooth smallest), other teeth relatively similar in size; mesobasal denticles present adjacent to the first-third proximal teeth, numbering more than 20; dorsally bearing a band of bristles that becomes broader and denser from proximal to the distal end; distal bristles not forming a typical rastellum. Maxillae (Fig. 12E) possessing distinct anterior lobe and posterior heel. Cuspules on maxillae and labium peg-like, notably smaller, more tenuous, and less distinct compared to those of females; the number of cuspules on maxillae shows three count ranges: 60 in KYUARA#1531 (the smallest individual), 70–76 in normal-sized individuals, and 80–85 in KYUARA#1509 (the largest individual); labium bearing only 2 or 3 cuspules. Three pairs of sternal sigilla (Fig. 12G), becoming closer to outer margin of sternum from posterior to anterior; posterior pair distinctly larger than anterior two pairs; anterior two pairs nearly equal in size; anteriormost pair almost merging into outer margin of sternum. All sternal sigilla more sclerotized and with more defined margins compared to the male of *D. dai* sp. nov. (Fig. 12I). Opisthosoma (Fig. 12H) oval. Four spinnerets (*n* = 7): PMS: 0.69 ± 0.05 long, 0.26 ± 0.02 diameter, PLS: 2.19 ± 0.08 long, 0.50 ± 0.05 maximal diameter, length of basal, medial, and apical segments 1.06 ± 0.05, 0.53 ± 0.04, 0.63 ± 0.05, respectively. Apical segment digitiform. Variations: sternum of the Yingjiang population is darker in color than that of the Lianghe population, and in this population all three pairs of sigilla are paler than the surrounding sternum.

**Figure 13.**
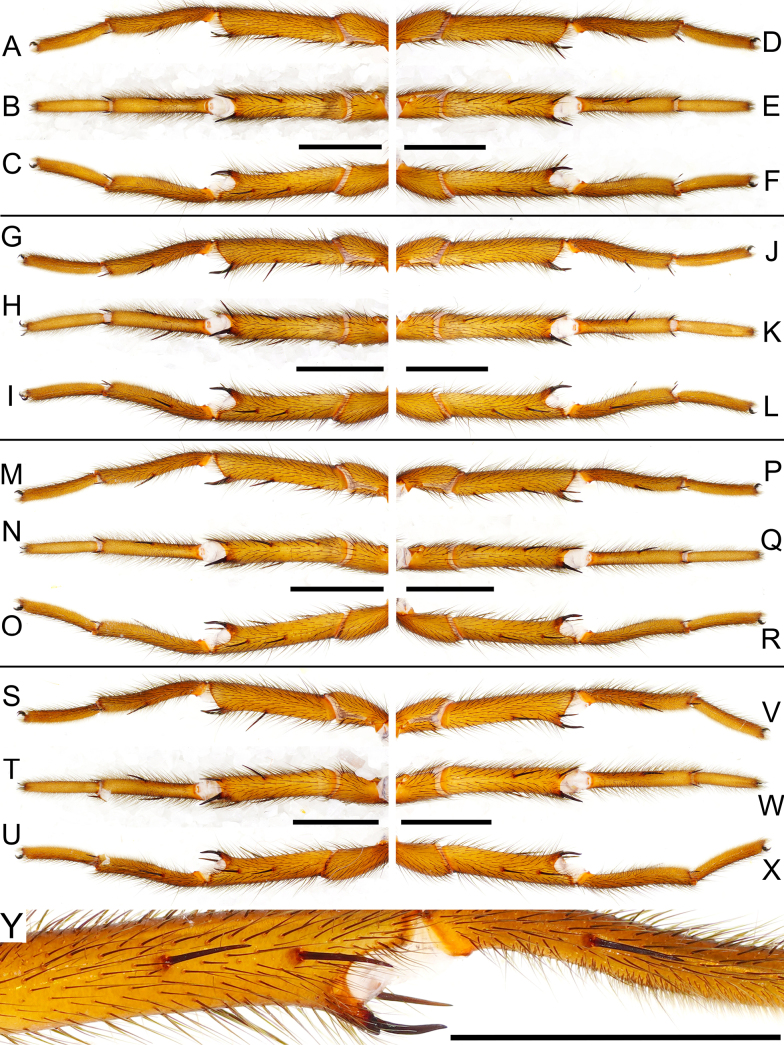
*Damarchus erebus* sp. nov. from Lianghe population, male secondary sexual characteristics on legs I, holotype (KYUARA#1509, **S–X**), paratype (KYUARA#1495, **A–F**), paratype (KYUARA#1496, **G–L**), paratype (KYUARA#1504, **M–R**). **A–C, G–I, M–O, S–U**. Left sides; **D–F, J–L, P–R, V–X**. Right sides; **Y**. Holotype left tibial spur, prolateral view; **C, F, I, L, O, R, U, X**. Prolateral; **B, E, H, K, N, Q, T, W**. Ventral; **A, D, G, J, M, P, S, V**. Retrolateral. Scale bars: 3 mm.

Spination of anterior legs (including large spine-like bristles; Figs 13, 18). Based on homologous structures across different population individuals, the most frequently observed arrangement is used for description. General: Leg I, femur pd (0–0–1); tibia pl (–1–1–), pv (0–0–Sp), rv (1–1–1); metatarsus pl (0–1–0), pv (0–0–1), rv (1–1–1). Leg II, femur pd (0–1–1); patella pd (0–0–1); tibia pl (–1–1–), pv (0–0–1), rv (1–1–1); metatarsus pl (0–1–0), pv (0–0–1), rv proximal spine weakest or absent, expressed as (0/1–1–1). Variations (due to too many of individuals and left-right asymmetry, only differences among populations are compared): Lianghe population (Fig. 13), rv proximal spine weakest or absent, expressed as (0/1–1–1) on both metatarsi I; Yingjiang population (Fig. 18), with additional pd (0–0–1) and Rv (0–0–1) on both patellae I, pv as (0–1–Sp) on both tibiae I; pd as (1–1–1) on both femora II, pd as (1–1–1) on both patellae II.

Leg scopulae. On tarsi I–IV and distal half of metatarsi I and II.

Trichobothria. Generally, 15–24 in two rows on tibiae, 12–23 in one row on metatarsi, 11–15 in one zigzag row on tarsi, respectively.

Metatarsal preening combs. On II–IV; II with Pv comb only, close to base of Pv spine (weakly developed, resembling bristles or inconspicuous); III–IV with both Pv and Rv combs, close to base of Pv and Rv spines, respectively; each comb commonly composed of three teeth (short spines). Variations: two teeth on all combs on III and Pv comb on IV of KYUARA#1531; four teeth on Rv combs on IV of KYUARA#1539–1540; four teeth on Pv combs on III and four teeth on Rv combs on IV KYUARA#1495 and KYUARA#1509.

Claws with ~ 8 teeth in one S-shaped row on all paired claws, proximal tooth smallest. Unpaired claw present on all tarsi and edentate.

Secondary sexual characteristics (Figs 13Y, 18G). Coupling spur on distal part of tibia I, with one stout spine (7–8 × longer than wide) distally. Sub-proximal portion of metatarsus I curved in lateral view; patch of short dense setae developing on proximal one-third to half portion of Pv side of metatarsus I.

Palp (Figs 15, 20A–C). Tibia with two strong bristles near Pv side, with a retrolateral bump on Rl side. Tegulum bulbous, large, and wide; embolus short, with length approximately equal to the width of the tegulum, gradually tapering, slightly curved at apex; no laminae or ribs on lower side, no para-embolic apophysis. Variations (Fig. 16F–H): In Lianghe population tegula are peach-shaped with strongly curved lateral margins, whereas in Yingjiang population lateral margins of tegula are more gently curved, appearing nearly as a pair of parallel lines.

##### Description of female.

KYUARA#1514. Coloration. In ethanol (Fig. 12B, F, I–J) carapace and chelicerae dark; palp and legs light brown, femora slightly darker than remaining segments; opisthosoma dark brown dorsally, with pale spots; paler ventrally, with almost no brown spots; book lung covers pale yellowish.

Measurements. Lianghe population (*n* = 5): Total length (excluding chelicerae): 13.17 ± 0.82 (12.35–14.35) (including paratypes: KYUARA#1505–1506 and KYUARA#1513–1515). Carapace 6.19 ± 0.55 long, 4.99 ± 0.32 wide. Eyes (Fig. 12D). Sizes and interdistances: AME 0.22 ± 0.02, ALE 0.36 ± 0.03, PLE 0.32 ± 0.03, PME 0.24 ± 0.03, AME–AME 0.16 ± 0.02, AME–ALE 0.15 ± 0.02, ALE–PLE 0.10 ± 0.04, PLE–PME 0.06 ± 0.03, PME–AME 0.15 ± 0.02, PME–PME 0.55 ± 0.05; eye group 0.65 ± 0.06 long, wide anteriorly 1.31 ± 0.10, posteriorly 1.36 ± 0.10; MOA 0.59 ± 0.05 long, wide anteriorly 0.57 ± 0.05, posteriorly 0.88 ± 0.08. Labium (Fig. 12F) 1.20 ± 0.12 long, 0.74 ± 0.01 wide. Maxillae (Fig. 12F) 2.57 ± 0.24 long, 1.33 ± 0.07 wide. Sternum (Fig. 12I) 3.53 ± 0.25 long, 3.19 ± 0.23 wide. For measurements of palp and legs see Table 7.

**Table 7.** *Damarchus erebus* sp. nov., Lianghe population, female palp, and legs measurements (*n* = 5).

**Segment**	**Femur**	**Patella**	**Tibia**	**Metatarsus**	**Tarsus**	**Total**
**Appendage**						
**Palp**	3.05 ± 0.16 (2.85–3.20)	1.65 ± 0.13 (1.43–1.78)	1.82 ± 0.12 (1.67–2.02)		1.65 ± 0.13 (1.43–1.78)	**1.82 ± 0.12 (1.67–2.02)**
**Leg I**	4.25 ± 0.33 (3.90–4.75)	2.49 ± 0.21 (2.20–2.76)	2.88 ± 0.21 (2.65–3.17)	2.36 ± 0.18 (2.15–2.58)	1.74 ± 0.08 (1.64–1.85)	**13.72 ± 0.82 (12.91–14.85)**
**Leg II**	3.86 ± 0.24 (3.50–4.18)	2.38 ± 0.13 (2.20–2.55)	2.40 ± 0.13 (2.20–2.57)	2.21 ± 0.31 (1.94–2.73)	1.71 ± 0.12 (1.56–1.90)	**12.56 ± 0.67 (11.58–13.58)**
**Leg III**	3.32 ± 0.24 (3.08–3.70)	2.08 ± 0.21 (1.73–2.30)	1.68 ± 0.13 (1.53–1.90)	2.40 ± 0.17 (2.26–2.68)	1.80 ± 0.13 (1.62–1.96)	**11.32 ± 0.51 (10.53–11.86)**
**Leg IV**	4.63 ± 0.38 (4.20–5.10)	2.45 ± 0.18 (2.33–2.75)	3.09 ± 0.23 (2.84–3.40)	3.71 ± 0.32 (3.34–4.10)	1.87 ± 0.11 (1.73–1.98)	**15.76 ± 0.86 (14.92–16.89)**

Measurements. Yingjiang population (KYUARA#1526, this specimen died due to dehydration, after that immersed in ethanol, Fig. 17B). Total length (excluding chelicerae): 9.75. Carapace 4.96 long, 4.50 wide. Eyes (Fig. 17D). Sizes and interdistances: AME 0.20, ALE 0.28, PLE 0.26, PME 0.19, AME–AME 0.12, AME–ALE 0.13, ALE–PLE 0.08, PLE–PME 0.03, PME–AME 0.09, PME–PME 0.44; eye group 0.55 long, wide anteriorly 1.08, posteriorly 1.12; MOA 0.47 long, wide anteriorly 0.50, posteriorly 0.74. For measurements of palp and legs see Table 8.

**Table 8.** *Damarchus erebus* sp. nov., Yingjiang population, female palp, and legs measurements (*n* = 1).

**Segment**	**Femur**	**Patella**	**Tibia**	**Metatarsus**	**Tarsus**	**Total**
**Appendage**						
**Palp**	2.56	1.45	1.65		1.63	**7.29**
**Leg I**	3.67	2.08	2.47	2.00	1.50	**11.72**
**Leg II**	3.28	2.00	2.16	1.92	1.46	**10.82**
**Leg III**	2.82	1.80	1.36	2.08	1.45	**9.51**
**Leg IV**	3.80	1.98	2.63	3.20	1.62	**13.23**

Carapace (Fig. 12B) relatively smooth, bearing narrow membranous region laterally on its outer margin, near coxae III. Almost no setae on carapace, setae generally sparse along outer margin of carapace, bristles on cephalic region; those on cephalic region forming one median longitudinal row. Cephalic region obviously raised. Eyes (Fig. 12B, D) on low mound, PME, PLE whiter than AME, AME slightly whiter than ALE. Fovea (Fig. 12B) procurved, width ~ 1/2 width of the ocular area. Chelicerae. Paturon (Fig. 12F) with eight or nine promarginal teeth in one row (the ninth tooth smallest), other teeth relatively similar in size; mesobasal denticles present adjacent to the first-third proximal teeth, numbering more than 20; dorsally bearing band of bristles that becomes broader and denser from proximal to distal end; distal bristles not forming a typical rastellum. Maxillae (Fig. 12F) possessing distinct anterior lobe and posterior heel. Cuspules on maxillae and labium peg-like, notably bigger, more distinct, stouter, and more strongly sclerotized compared to those of males; the number of cuspules on maxillae shows three count ranges: 55–60 KYUARA#1526 (the smallest individual), 76–80 KYUARA#1515 (a slightly larger than KYUARA#1526 individual), and 105–120 in normal-sized individuals; labium bearing two or three cuspules. Three pairs of sternal sigilla (Fig. 12I), become closer to outer margin of sternum from posterior to anterior; posterior pair distinctly larger than anterior two pairs; anterior two pairs nearly equal in size; anteriormost pair almost merging into outer margin of sternum. All sternal sigilla more sclerotized and with more defined margins compared to those of males (Fig. 12G). Opisthosoma (Fig. 12J) oval. Four spinnerets (*n* = 6): PMS: 0.81 ± 0.05 long, 0.34 ± 0.04 diameter, PLS: 2.33 ± 0.16 long, 0.62 ± 0.02 maximal diameter, length of basal, medial, and apical segments 1.20 ± 0.05, 0.51 ± 0.04, 0.57 ± 0.04, respectively. Apical segment digitiform.

**Figure 14.**
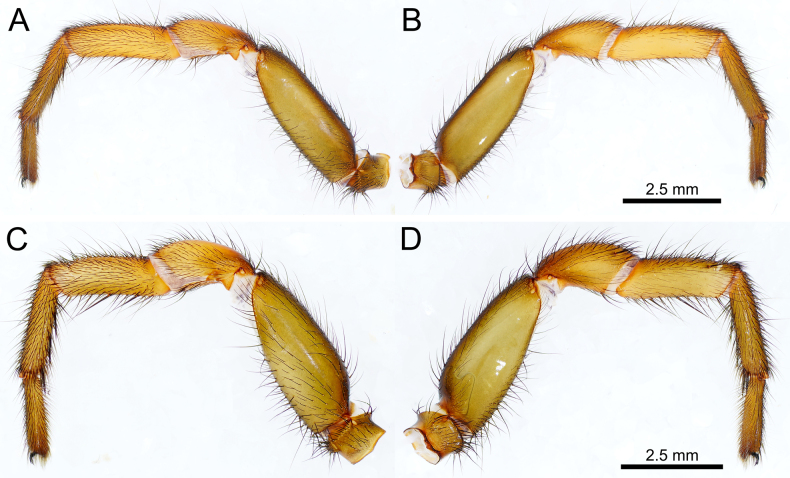
*Damarchus erebus* sp. nov. from Lianghe population, female (KYUARA#1514) paratype legs. **A, B**. Legs I; **C, D**. Legs II; **A, C**. Retrolateral; **B, D**. Prolateral.

Spination of anterior legs (including large spine-like bristles; Figs 14, 19). General: Leg I femur pd (0–0–1); tibia rv (1–1–0); metatarsus pv (0–0–1), rv (1–1–1). Leg II femur pd (0–0–1); patella pd (0–1–1); tibia rv (1–1–0); metatarsus pv (0–0–1), rv (1–1–1). Variations: Lianghe population, with additional pd (0–0–1) on both tibiae II. Yingjiang population, with additional pl (0–1–0) on both tibiae II and metatarsi II.

**Figure 15.**
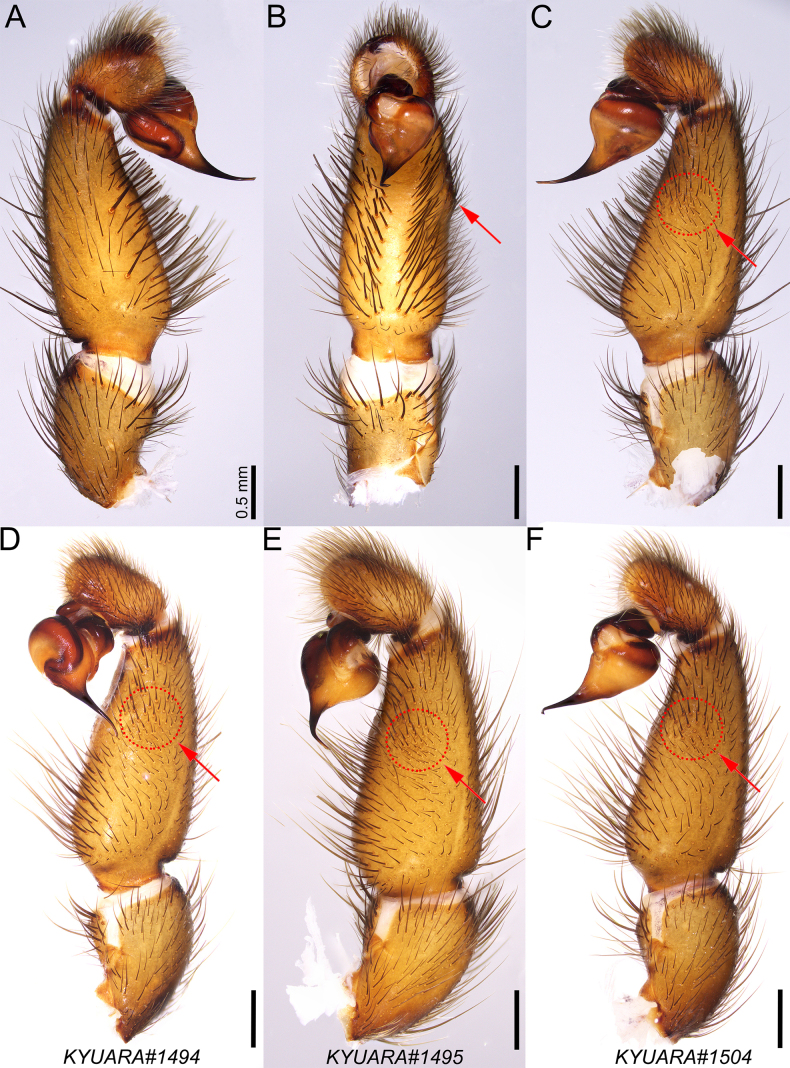
*Damarchus erebus* sp. nov. from Lianghe population, male palps of holotype (**A–C**) and paratypes (**D–F**). **A**. Prolateral; **B**. Ventral; **C–F**. Retrolateral.

**Figure 16.**
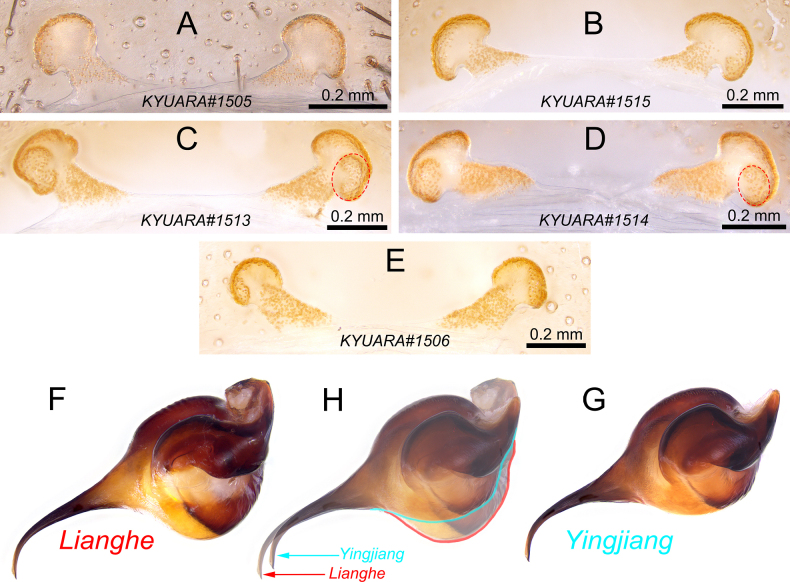
*Damarchus erebus* sp. nov. from Lianghe population. **A–E**. Spermathecae of paratypes; **F**. Palpal bulb of Lianghe population; **G**. Palpal bulb of Yingjiang population; **H**. Palpal bulb comparison between the Lianghe population and the Yingjiang population; **F–H**. Retrolateral; Red (Lianghe population); blue (Yingjiang population).

**Figure 17.**
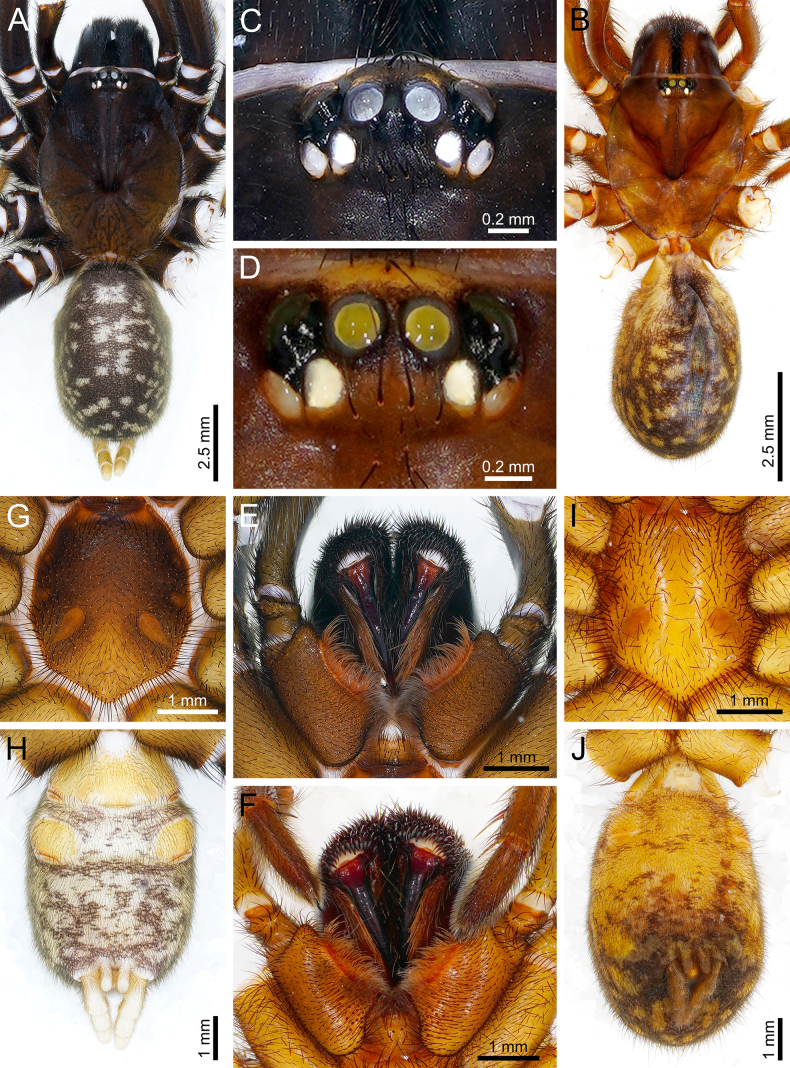
*Damarchus erebus* sp. nov. from Yingjiang population, male (KYUARA#1540, **A, C, E, G, H**) and female (KYUARA#1526, **B, D, F, I–J**). **A, B**. Habitus; **C, D**. Ocular area; **E, F**. Chelicerae and maxillae; **G, I**. Sternum; **H, J**. Opisthosoma. **A–D**. Dorsal; **E–J**. Ventral.

**Figure 18.**
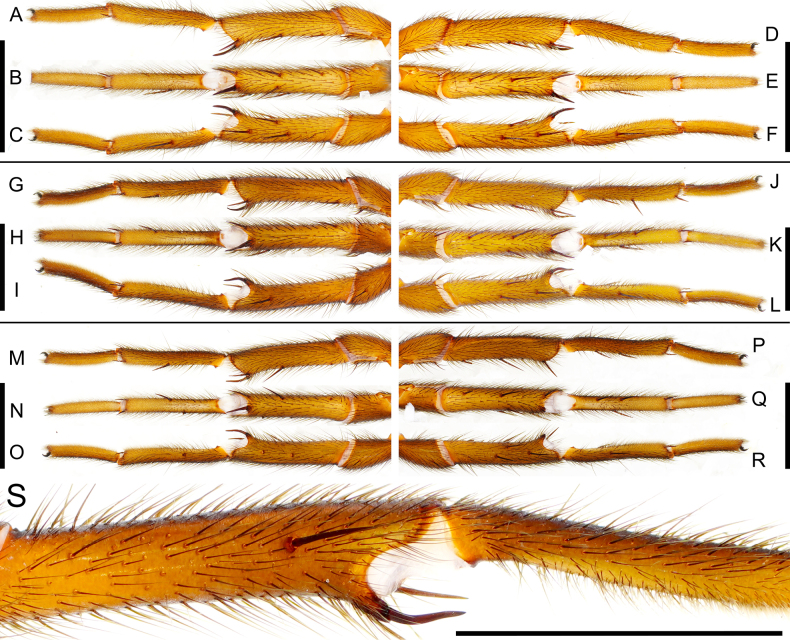
*Damarchus erebus* sp. nov. from Yingjiang population, male secondary sexual characteristics on legs I, KYUARA#1531 (**A–F**), KYUARA#1539 (**G–L**), KYUARA#1540 (M**–**R). **A–C, G–I, M–O**. Left sides; **D–F, J–L, P–R**. Right sides; **S**. KYUARA#1539 left tibial spur, prolateral view; **C, F, I, L, O, R**. Prolateral; **B, E, H, K, N, Q**. Ventral; **A, D, G, J, M, P**. Retrolateral. Scale bars: 3 mm.

**Figure 19.**
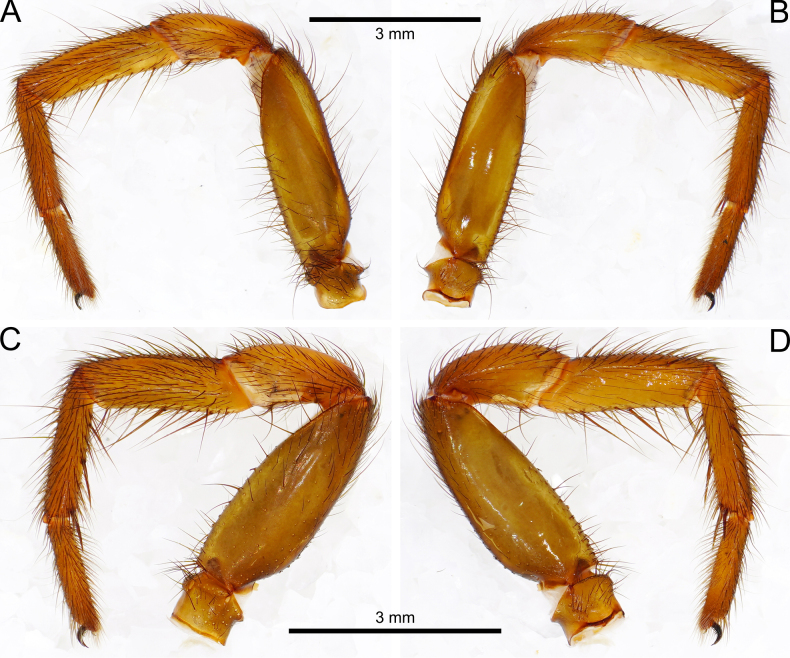
*Damarchus erebus* sp. nov. from Yingjiang population, female (KYUARA#1526) legs. **A, B**. Legs I; **C, D**. Legs II. **A, C**. Retrolateral; **B, D**. Prolateral.

**Figure 20.**
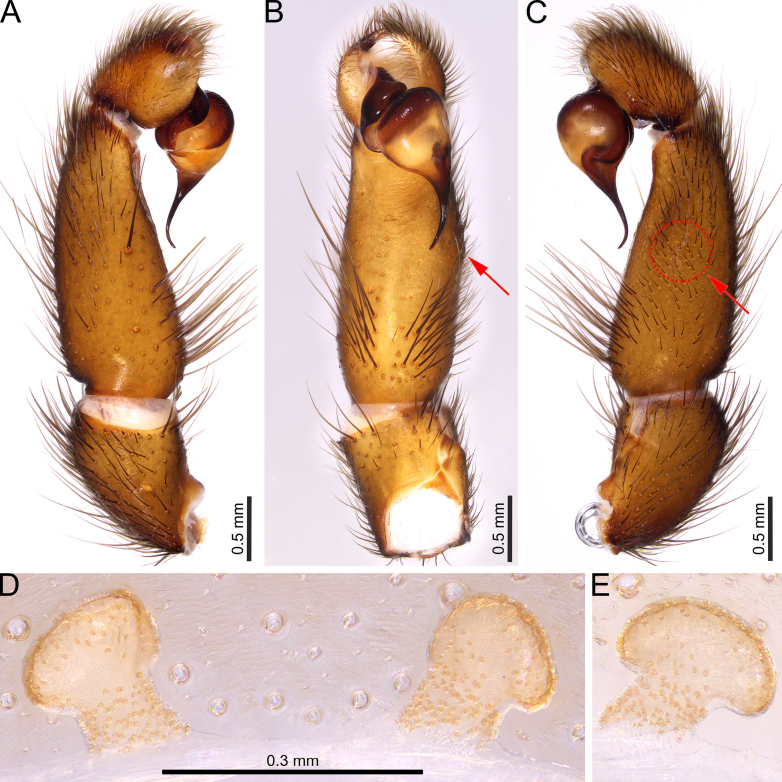
*Damarchus erebus* sp. nov. from Yingjiang population, male (KYUARA#1540) palps (**A–C**) and female (KYUARA#1526) spermathecae (**D–E**). **A**. Prolateral; **B**. Ventral; **C**. Retrolateral.

**Figure 21.**
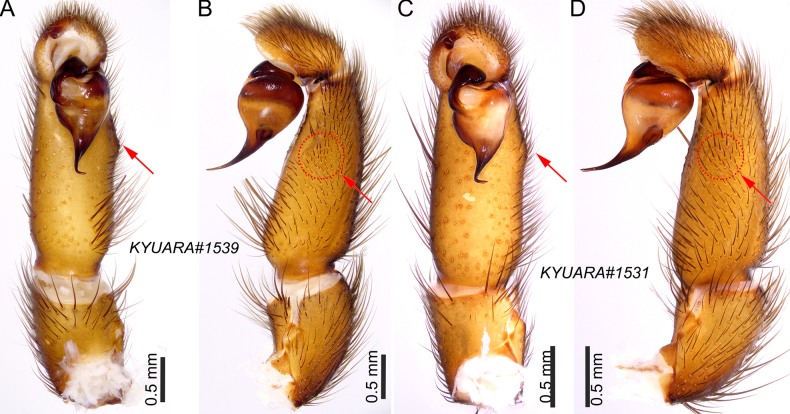
*Damarchus erebus* sp. nov. from Yingjiang population, male palps of KYUARA#1539 (**A, B**), KYUARA#1531 (**C, D**). **A, C**. Ventral; **B, D**. Retrolateral.

Leg scopulae. On tarsi I–IV and distal half of metatarsi I and II.

Trichobothria. Paratype, 18–22 in two rows on tibiae, 14–18 in one row on metatarsi, 11–14 in one zigzag row on tarsi. Variations: Yingjiang population, 14–16 on tibiae I–IV.

Metatarsal preening combs. On legs II–IV; II with Pv comb only, close to base of Pv spine; III–IV with both Pv and Rv combs, close to base of Pv and Rv spines, respectively; each comb commonly composed of 3 teeth (short spines). Variations: Yingjiang population, 4 teeth on Pv comb on metatarsi III.

Claws. 2 rows of teeth, ~ 4–6 teeth per row on outer claws and 4 teeth per row on inner claws on all paired claws, proximal tooth smallest. Unpaired claw present on all tarsi and edentate. Variations: Yingjiang population, 6 teeth on both outer and inner claws.

Spermathecae (Figs 16A–E, 20D–E). More or less mushroom-shaped, distinctly everted. Stalk short, similar in length to the receptacle, densely covered with glandular pores, broad at base, constricting markedly toward upper 1/2; receptacle with glandular pores only on uppermost surface. Variations: KYUARA#1513–1514, receptacle with a retrolateral bump (indicated by red circle, Fig. 16D–E); KYUARA#1506, receptacle-to-stalk length ratio greater than that in other specimens.

##### Etymology.

The specific epithet refers to *Erebus* (Ἔρεβος), one of the primordial deities in Greek mythology, representing darkness and the underworld. Noun in apposition.

##### Remarks.

All examined specimens were collected from Dehong and are considered here as belonging to two geographical populations: the Lianghe population (type series, 4 ♂, 5 ♀ examined) and the Yingjiang population (non-type specimens, 3 ♂ 1 ♀ examined). The *COI* genetic distance (Table 13) within the Lianghe population ranges from 0.4–2.4% (mean distances ~ 1.58%), while distance within the Yingjiang population ranges from 0.3–2.7% (mean distances ~ 1.9%). A relatively significant difference is observed between the Lianghe and Yingjiang populations, with a genetic distance reaching 13.3–14.3% (mean distances ~ 13.7%). However, no reliable morphological differences were identified between these two populations based on the available specimens, particularly in the key sexual features (i.e., female vulva, male palpal organ and coupling spur). The tegulum of individuals from Lianghe population appears slightly larger than that of Yingjiang population (Fig. 16F–H), which is stable based on comparisons of multiple population’s individuals. However, spermathecae of the two populations show no difference, this may be related to the fact that only a single female specimen is available from Yingjiang population.

Given the lack of identifiable morphological distinctions, the Lianghe and Yingjiang populations are here treated as conspecific within the current taxonomy of *Damarchus*, rather than being separated based solely on the currently available molecular data.

#### Damarchus rutilus sp. nov.

Taxon classificationAnimaliaAraneaeBemmeridae

A806F665-CB92-5508-8E43-81FC65B262DB

https://zoobank.org/DFBD08DA-3F1D-4E10-B32E-54BF9EE2C97D

Figs 22, 23, 24, 25, 26, 27, 28, 29, 30, 31, Tables 9–12

##### Chinese name.

橙达玛蛛.

##### Type material.

***Holotype***: China • ♂; Yunnan Province, Dehong Dai and Jingpo Autonomous Prefecture, Yingjiang County, TPNR; 24.66807°N, 97.598961°E, alt. 913.4 m, 5 Oct. 2024; K. Yu, Y. Ni & Y. Xu leg.; GenBank: PX967114; KYUARA#1534, HBUARA#2024-882. ***Paratypes***: China • 1 ♂; same data as holotype also TPNR; Apr. 2023 (matured Oct. 2023); Z. Li leg.; GenBank: PX967113; KYUARA#1530. • 1 ♂; same data as preceding, but 24.717877°N, 97.574119°E, alt. 245.40 m, 7 Oct. 2024; K. Yu, Y. Ni & Y. Xu leg.; GenBank: PX967115; KYUARA#1535, HBUARA#2024-883. • 1 ♂; same data as preceding; Apr. 2023; Z. Li. leg.; GenBank: PX967116; KYUARA#1575. • 1 ♀; same data as preceding; Apr. 2023; GenBank: PX967117; KYUARA#1528.

##### Other material examined.

China • 1 ♂ 1 ♀; same data as preceding but, Lianghe County, Kazi Village; 24.601667°N, 98.239444°E; male matured 23 Jun. 2021, S. Zhang leg.; GenBank: PX967111, KYUARA#1507; GenBank: PX967112, KYUARA#1508.

##### Diagnosis.

*D. rutilus* sp. nov. shares a similar overall appearance with *D. inazuma* in following characters: (1) living male specimens covered with a whitish frosted coating on both the carapace and the legs; (2) in ethanol, the appearance and coloration of all body parts are similar in both male and female. (3) the male carapace bears dense pubescence that entirely covers its surface; the tegulum approximately tulip-bulb shape, with a slender and elongated embolus, gradually tapering, its apex very thin and slightly curved. Moreover *D. rutilus* sp. nov. can be distinguished from *D. inazuma* by following characters: (1) the presence of one stout spine (7–8 × longer than wide) distally at the tip of the coupling spur on leg I (vs three straight adjacent spines, grouped in a single row in *D. inazuma*); (2) the slightly everted spermathecal stalk (vs spermathecal stalk incurved in *D. inazuma*).

##### Description of male.

**Figure 22.**
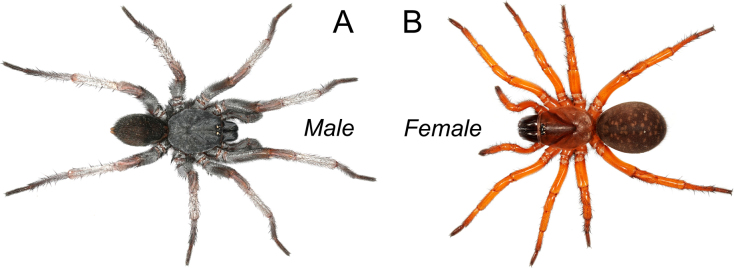
*Damarchus rutilus* sp. nov., living spiders from Yingjing population. **A**. Male holotype (KYUARA#1534); **B**. Female paratype (KYUARA#1528).

**Figure 23.**
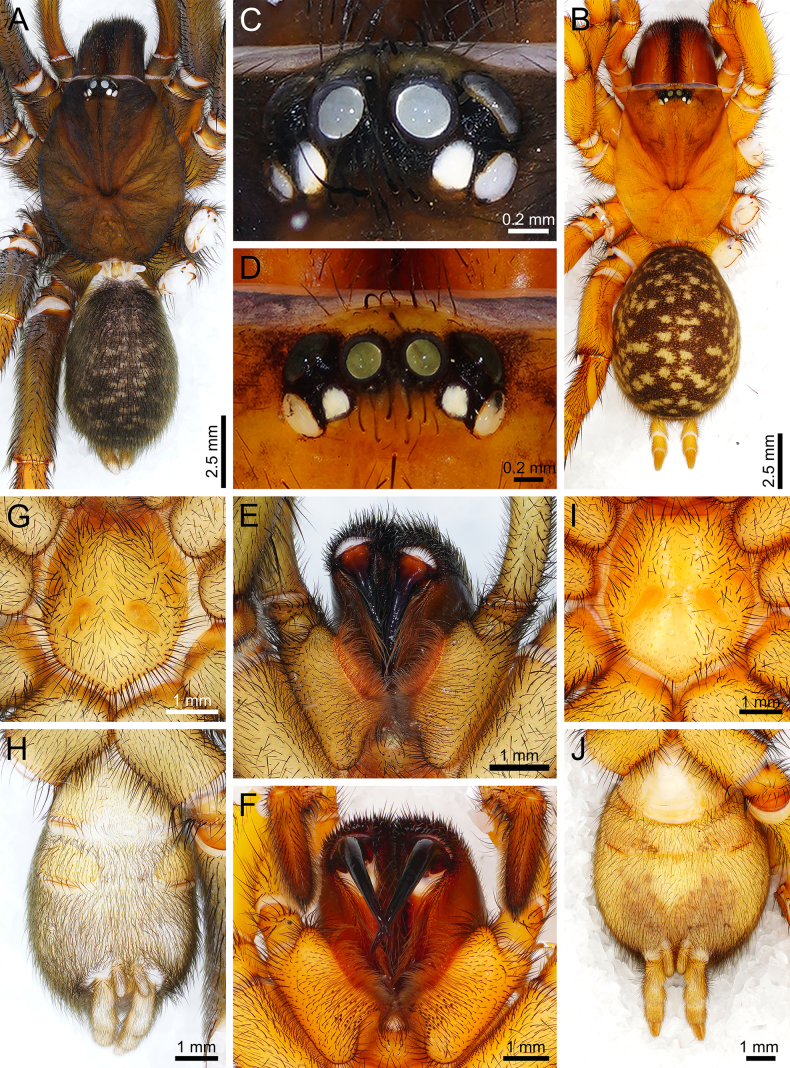
*Damarchus rutilus* sp. nov. from Yingjing population, male holotype (**A, C, E, G, H**) and female paratype (**B, D, F, I–J**). **A, B**. Habitus; **C, D**. Ocular area; **E, F**. Chelicerae and maxillae; **G, I**. Sternum; **H, J**. Opisthosoma; **A–D**. Dorsal; **E–J**. Ventral.

KYUARA#1534. Coloration. Living spiders (Fig. 22A) carapace and chelicerae dark; carapace, femora and tibiae covered with whitish frosted coating; palp and femora slightly darker than remaining segments; opisthosoma dark brown dorsally, paler ventrally, with some brown setae; book lung covers pale yellowish. In ethanol (Fig. 23A, E, G–H), color slightly lighter than in life, whitish frosted coating not obvious, opisthosoma dorsal with pale spots.

Measurements. Yingjiang population (*n* = 4): Total length (excluding chelicerae): 11.29 ± 0.84 (10.06–12.13) (including holotype KYUARA#1534, paratypes: KYUARA#1530, KYUARA#1535 and KYUARA#1575). Carapace 6.82 ± 0.61 long, 5.57 ± 0.42 wide. Eyes (Fig. 23C). Sizes and interdistances: AME 0.26 ± 0.04, ALE 0.40 ± 0.03, PLE 0.29 ± 0.03, PME 0.24 ± 0.02, AME–AME 0.17 ± 0.04, AME–ALE 0.09 ± 0.03, ALE–PLE 0.06 ± 0.01, PLE–PME 0.04 ± 0.01, PME–AME 0.09 ± 0.03, PME–PME 0.54 ± 0.04; eye group 0.64 ± 0.01 long, wide anteriorly 1.27 ± 0.09, posteriorly 1.31 ± 0.11; MOA 0.57 ± 0.01 long, wide anteriorly 0.64 ± 0.07, posteriorly 0.89 ± 0.05. Labium (Fig. 23E) 1.35 ± 0.19 long, 0.68 ± 0.08 wide. Maxillae (Fig. 23E) 2.50 ± 0.22 long, 1.10 ± 0.09 wide. Sternum (Fig. 23G) 3.55 ± 0.27 long, 2.98 ± 0.20 wide. For measurements of palp and legs see Table 9.Lianghe population (KYUARA#1507, Fig. 28A): Total length (excluding chelicerae) 14.20. Carapace 7.20 long, 5.80 wide. Eyes (Fig. 28C). Sizes and interdistances: AME 0.37, ALE 0.43, PLE 0.32, PME 0.21, AME–AME 0.16, AME–ALE 0.13, ALE–PLE 0.11, PLE–PME 0.05, PME–AME 0.07, PME–PME 0.60; eye group 0.71 long, wide anteriorly 1.33, posteriorly 1.38; MOA 0.70 long, wide anteriorly 0.74, posteriorly 0.92. For measurements of palp and legs see Table 10.

**Table 9.** *Damarchus rutilus* sp. nov., Yingjiang population, male palp, and legs measurements (*n* = 4).

**Segment**	**Femur**	**Patella**	**Tibia**	**Metatarsus**	**Tarsus**	**Total**
**Appendage**						
**Palp**	3.24 ± 0.21 (3.10–3.55)	1.66 ± 0.25 (1.28–1.90)	2.98 ± 0.14 (2.90–3.18)		1.17 ± 0.05 (1.13–1.23)	**9.05 ± 0.53 (8.52–9.76)**
**Leg I**	6.05 ± 0.28 (5.76–6.40)	3.15 ± 0.19 (3.04–3.43)	5.04 ± 0.28 (4.64–5.26)	4.58 ± 0.15 (4.37–4.70)	2.75 ± 0.09 (2.66–2.86)	**21.56 ± 0.91 (20.49–22.65)**
**Leg II**	5.51 ± 0.21 (5.26–5.76)	2.82 ± 0.28 (2.50–3.22)	4.47 ± 0.20 (4.16–4.62)	4.33 ± 0.14 (4.13–4.49)	2.60 ± 0.10 (2.48–2.70)	**19.72 ± 0.78 (18.83–20.70)**
**Leg III**	4.70 ± 0.10 (4.60–4.80)	2.29 ± 0.32 (1.82–2.55)	3.40 ± 0.06 (3.33–3.47)	4.67 ± 0.14 (4.48–4.80)	2.39 ± 0.10 (2.27–2.47)	**17.45 ± 0.44 (17.02–18.02)**
**Leg IV**	6.78 ± 0.25 (6.47–7.05)	2.69 ± 0.36 (2.28–3.15)	5.25 ± 0.20 (4.97–5.37)	7.15 ± 0.39 (6.63–7.50)	2.71 ± 0.11 (2.57–2.86)	**24.57 ± 1.07 (23.37–25.91)**

**Table 10.** *Damarchus rutilus* sp. nov., Lianghe population, male palp, and legs measurements (*n* = 1).

**Segment**	**Femur**	**Patella**	**Tibia**	**Metatarsus**	**Tarsus**	**Total**
**Appendage**						
**Palp**	3.32	1.42	2.90		1.24	**8.88**
**Leg I**	6.20	3.28	5.01	4.68	2.80	**21.97**
**Leg II**	5.70	2.46	4.57	4.50	2.80	**20.03**
**Leg III**	4.20	2.50	3.28	4.73	2.55	**17.26**
**Leg IV**	6.98	3.15	5.20	7.28	2.75	**25.36**

Carapace (Fig. 23A) bearing dense scales almost entirely covering the surface, bristles relatively distinct along outer margin of the carapace, on cephalic region, and on posterior part of thoracic region; those on cephalic region forming one median longitudinal row. Cephalic region not obviously raised. Eyes (Fig. 23A, C) on low mound, posterior eyes noticeably whiter than the anterior eyes, AME whiter than ALE. Fovea (Fig. 23A) procurved, width ~ half to two-thirds width of the ocular area. Chelicerae. Paturon (Fig. 23E) with10–11 promarginal teeth in one row (the eleventh tooth smallest), other teeth relatively similar in size; mesobasal denticles present adjacent to the first-third proximal teeth, numbering more than 20; dorsally bearing band of bristles that becomes broader and denser from proximal to distal end; distal bristles not forming a typical rastellum. Maxillae (Fig. 23E) possessing distinct anterior lobe and posterior heel. Cuspules on maxillae and labium peg-like, notably smaller, more tenuous, and less distinct compared to those of females; the number of cuspules on maxillae shows variations among individuals: KYUARA#1534 30–33, KYUARA#1507 46–52, KYUARA#1530 56–58, KYUARA#1535 20–23, KYUARA#1575 40–43; labium bearing only two or three cuspules. Three pairs of sternal sigilla (Fig. 23G), become closer to outer margin of sternum from posterior to anterior; posterior pair distinctly larger than anterior two pairs; anterior two pairs nearly equal in size; anteriormost pair almost merging into outer margin of sternum. All sternal sigilla less sclerotized and with less defined margins compared to those of females (Fig. 23I). Opisthosoma (Fig. 23H) oval. Four spinnerets (*n* = 5): PMS: 0.87 ± 0.08 long, 0.29 ± 0.05 diameter, PLS: 2.67 ± 0.40 long, 0.60 ± 0.08 maximal diameter, length of basal, medial, and apical segments 1.25 ± 0.20, 0.63 ± 0.13, 0.80 ± 0.13, respectively. Apical segment digitiform.

**Figure 24.**
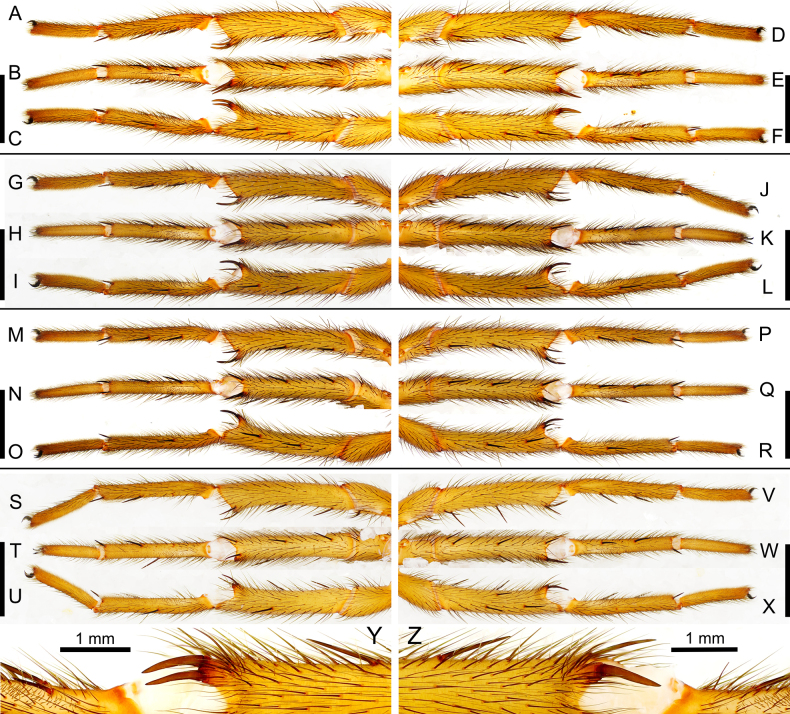
*Damarchus rutilus* sp. nov. from Yingjing population, male secondary sexual characteristics on legs I, holotype ((KYUARA#1534, **G–L**), paratype (KYUARA#1530, **A–F**), paratype (KYUARA#1535, **M–R**), paratype (KYUARA#1575, **S–X**). **A–C, G–I, M–O, S–U**. Left sides; **D–F, J–L, P–R, V–X**. Right sides; **Y**. Paratype (KYUARA#1530) left tibial spur, prolateral view. **Z**. Paratype (KYUARA#1530) right tibial spur, prolateral view; **C, F, I, L, O, R, U, X**. Prolateral; **B, E, H, K, N, Q, T, W**. Ventral; **A, D, G, J, M, P, S, V**. Retrolateral. Scale bars: 3 mm (**A–X**).

Spination of anterior legs (including large spine-like bristles; Figs 24, 29). General: Leg I, femur pd (0–0–1); tibia pl (–1–1–), pv (0–1–Sp), rv (1–1–1); metatarsus pl (0–1–0), pv (0–0–1), rv (1–1–1). Leg II, femur pd (0–0–1); patella pd (0–0–1); tibia pl (–1–1–), pv (0–0–2), rv (1–1–1); metatarsus pl (0–1–0), pv (0–0–1), rv (1–1–1). variations: Lianghe population (KYUARA#1507), pd as (–1–1–) on both femora I, pl as (1–1–1), pv as (0–1–Sp) on both tibiae I; pd as (–1–1–) on both femora II, rv as (1–1–2) on both metatarsi.

Leg scopulae. On tarsi I–IV and distal half of metatarsi I and II.

Trichobothria. Generally, 19–24 in two rows on tibiae, 18–22 in one row on metatarsi and 13–15 in one zigzag row on tarsi, respectively.

Metatarsal preening combs. On II–IV; II with Pv comb only, close to base of Pv spine (weakly developed, resembling bristles or inconspicuous); III–IV with both Pv and Rv combs, close to base of Pv and Rv spines, respectively; number of teeth per comb as follows: II 3/0, III 3–4/3–4, IV 3/4.

Claws. ~ 10–11 teeth in one S-shaped row on all paired claws, proximal tooth smallest. Unpaired claw present on all tarsi and edentate.

Secondary sexual characteristics (Figs 24I–J, 29C–D). Coupling spur on distal part of tibia I, with one stout spine (7–8 × longer than wide) distally. Sub-proximal portion of metatarsus I curved in lateral view; patch of short dense setae developing on proximal 1/3–1/2 portion of Pv side of metatarsus I. Variations: Paratype (KYUARA#1530) left coupling spur, two straight adjacent spines grouped in single row; only this individual exhibits this variation.

Palp (Figs 26A–C, 27, 31A–C). Tibia with two strong bristles near Pv side, lacks a retrolateral bump on Rl side. Tegulum bulbous, large. and wide, tulip-bulb shape; embolus slender, elongated, gradually tapering, with length ~ 2.5 × width of tegulum; no laminae or ribs on lower side, no para-embolic apophysis.

**Figure 25.**
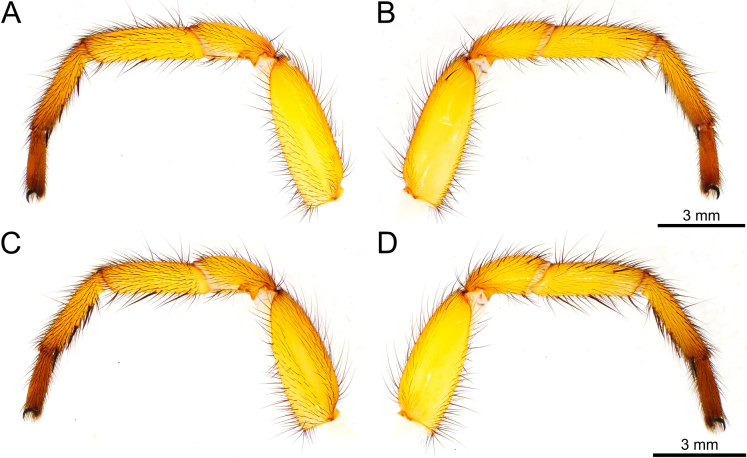
*Damarchus rutilus* sp. nov. from Yingjiang population, female paratype (KYUARA#1528) legs. **A, B**. Legs I; **C, D**. Legs II. **A, C**. Retrolateral; **B, D**. Prolateral.

**Figure 26.**
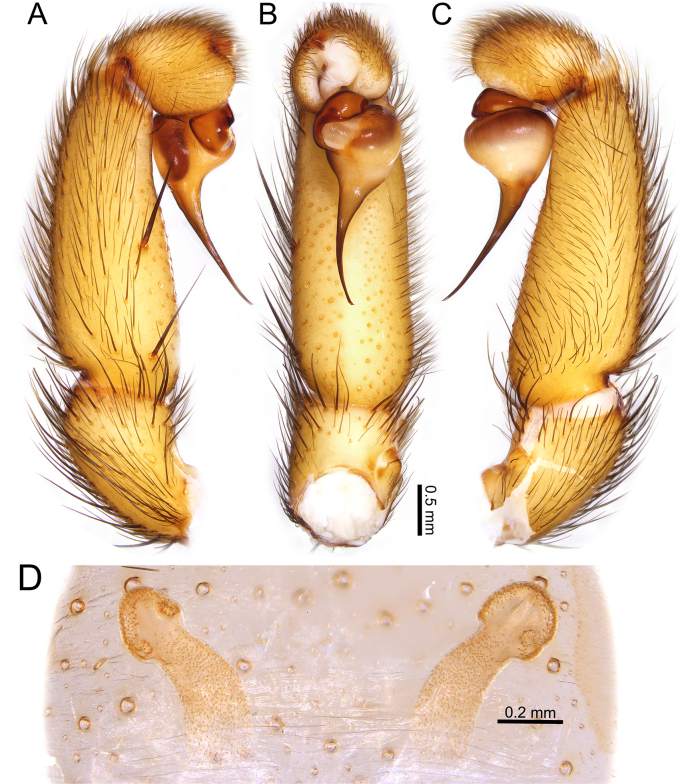
*Damarchus rutilus* sp. nov. from Yingjiang population, male holotype (KYUARA#1534) palps (**A–C**) and female paratype (KYUARA#1528) spermathecae (**D**). **A**. Prolateral; **B**. Ventral; **C**. Retrolateral.

**Figure 27.**
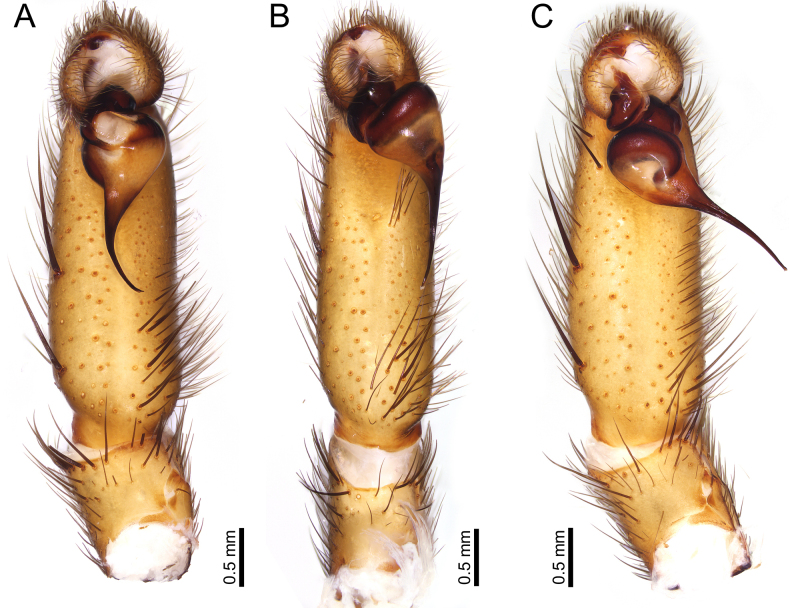
*Damarchus rutilus* sp. nov. from from Yingjiang population, male palps of paratypes: **A**. KYUARA#1530; **B**. KYUARA#1535; **C**. KYUARA#1575, ventral view.

##### Description of female.

KYUARA#1528. Coloration. Living spiders (Fig. 22B) chelicerae black; prosoma and appendages both orange-red, prosoma slightly darker than appendages, opisthosoma dark brown dorsally, with pale spots; paler ventrally, with brown setae, book lung covers pale orange. In ethanol (Fig. 23B, F, I–J), color slightly lighter than in life.

Measurements. Yingjiang population (paratype KYUARA#1528): Total length (excluding chelicerae): 13.51. Carapace 6.73 long, 5.52 wide. Eyes (Fig. 23D). Sizes and interdistances: AME 0.25, ALE 0.41, PLE 0.36, PME 0.26, AME–AME 0.17, AME–ALE 0.15, ALE–PLE 0.08, PLE–PME 0.08, PME–AME 0.11, PME–PME 0.45; eye group 0.69 long, wide anteriorly 1.47, posteriorly 1.50; MOA 0.58 long, wide anteriorly 0.62, posteriorly 0.97. Labium (Fig. 23F) 1.48 long, 0.92 wide. Maxillae (Fig. 23F) 2.85 long, 1.36 wide. Sternum (Fig. 23I) 3.85 long, 3.38 wide. For measurements of palp and legs see Table 11.

**Table 11.** *Damarchus rutilus* sp. nov., Yingjiang population, female palp, and legs measurements (*n* = 1).

**Segment**	**Femur**	**Patella**	**Tibia**	**Metatarsus**	**Tarsus**	**Total**
**Appendage**						
**Palp**	3.36	1.90	2.11		2.05	**9.42**
**Leg I**	4.95	3.00	3.60	2.90	1.92	**16.37**
**Leg II**	4.38	2.70	3.00	2.84	1.95	**14.87**
**Leg III**	3.83	2.35	2.30	2.91	1.74	**13.13**
**Leg IV**	5.73	2.96	3.85	5.06	2.08	**19.68**

**Figure 28.**
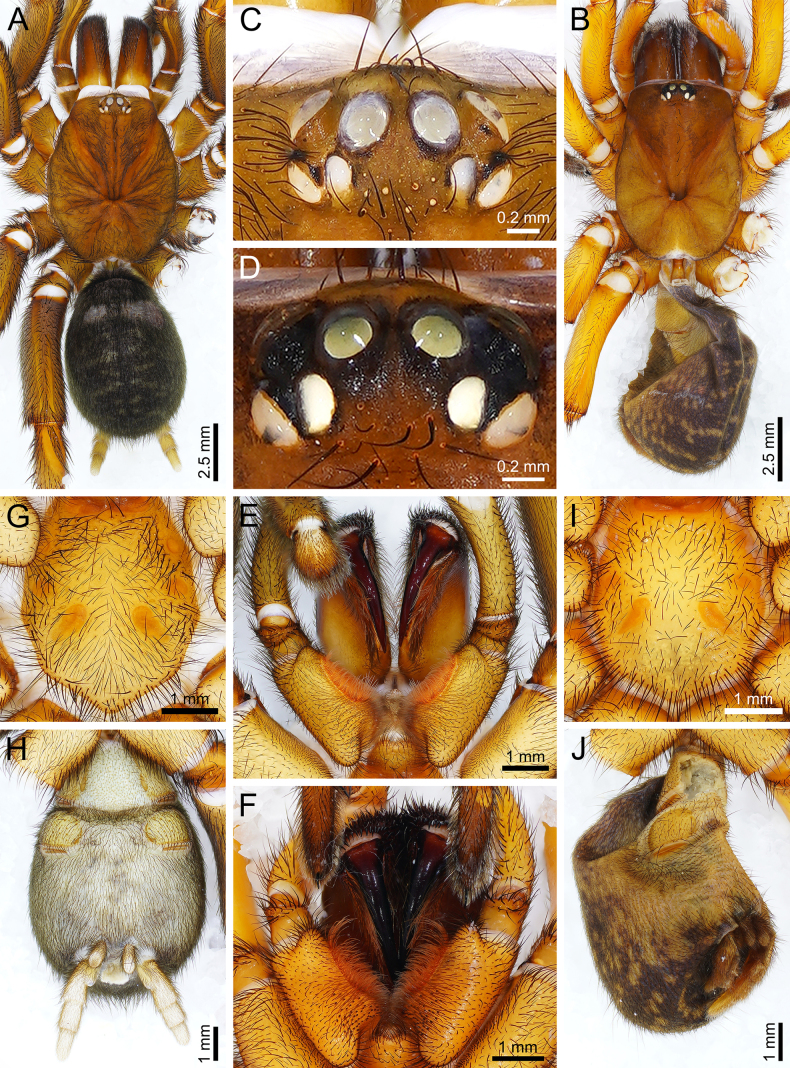
*Damarchus rutilus* sp. nov. from Lianghe population, male (KYUARA#1507, **A, C, E, G, H**) and female (KYUARA#1508, **B, D, F, I–J**). **A, B**. Habitus; **C, D**. Ocular area; **E, F**. Chelicerae and maxillae; **G, I**. Sternum; **H, J**. Opisthosoma. **A–D**. Dorsal; **E–J**. Ventral.

Lianghe population (KYUARA#1508): opisthosoma severely deformed, and therefore not measured. Carapace 6.90 long, 5.08 wide. Eyes (Fig. 28D). Sizes and interdistances: AME 0.32, ALE 0.42, PLE 0.31, PME 0.26, AME–AME 0.20, AME–ALE 0.09, ALE–PLE 0.07, PLE–PME 0.08, PME–AME 0.13, PME–PME 0.53; eye group 0.70 long, wide anteriorly 1.38, posteriorly 1.36; MOA 0.65 long, wide anteriorly 0.73, posteriorly 0.87. For measurements of palp and legs see Table 12.

**Figure 29.**
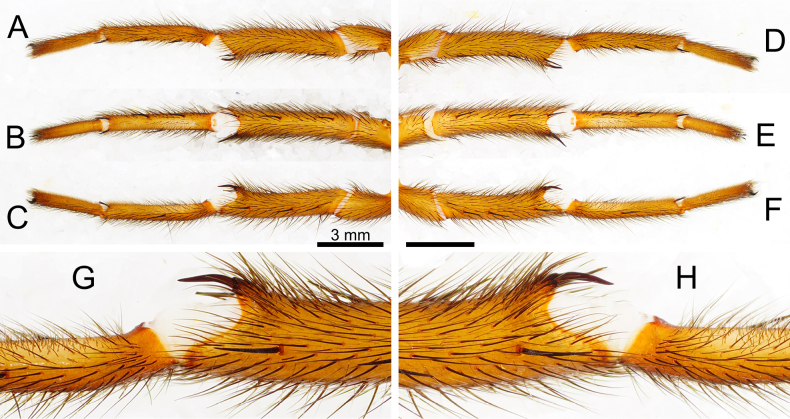
*Damarchus rutilus* sp. nov. from Lianghe population, male (KYUARA#1507) secondary sexual characteristics on legs I. **A–C, G**. Left side; **D–F, H**. Right side; **A, D**. Prolateral; **B, E**. Ventral; **C, F–H**. Retrolateral.

**Figure 30.**
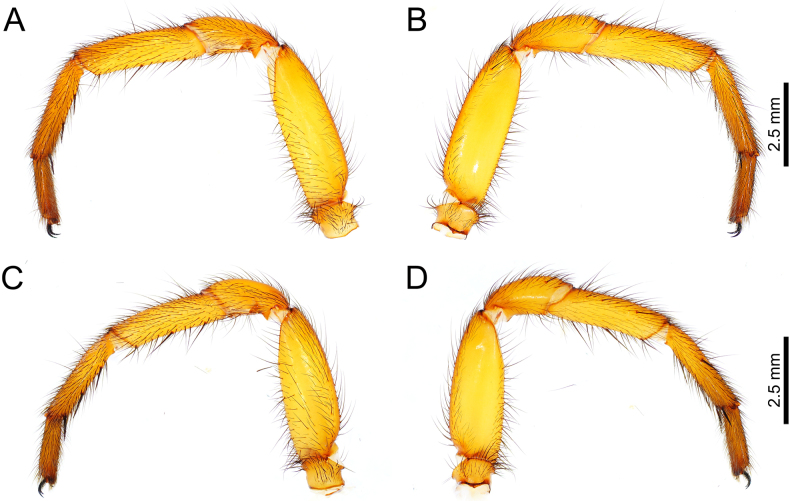
*Damarchus rutilus* sp. nov. from Lianghe population, female (KYUARA#1508) legs. **A, B**. Legs I; **C, D**. Legs II; **A, C**. Retrolateral; **B, D**. Prolateral.

**Figure 31.**
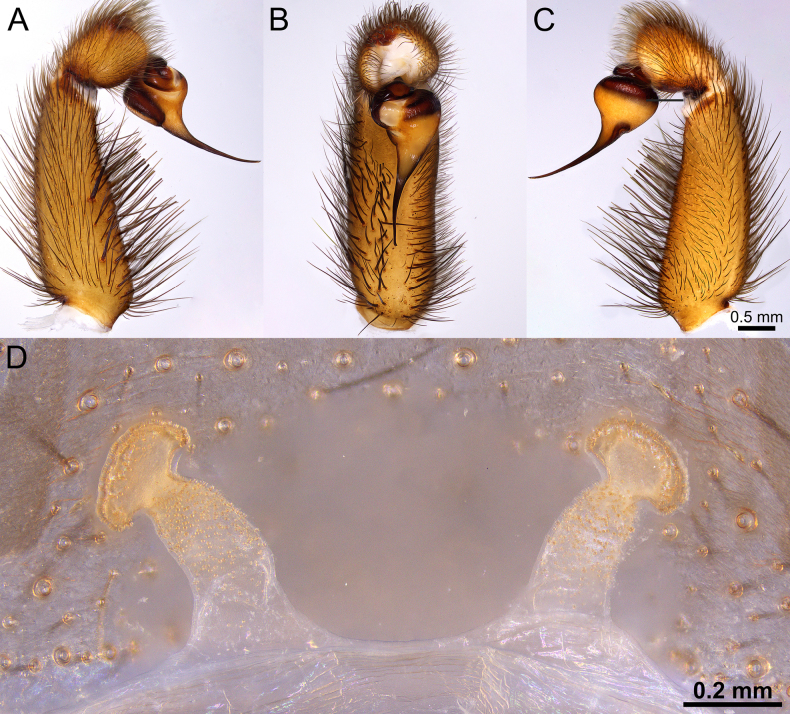
*Damarchus rutilus* sp. nov. from Lianghe population, male (KYUARA#1507) palps (**A–C**) and female (KYUARA#1508) spermathecae (**D**). **A**. Prolateral; **B**. Ventral; **C**. Retrolateral.

**Figure 32.**
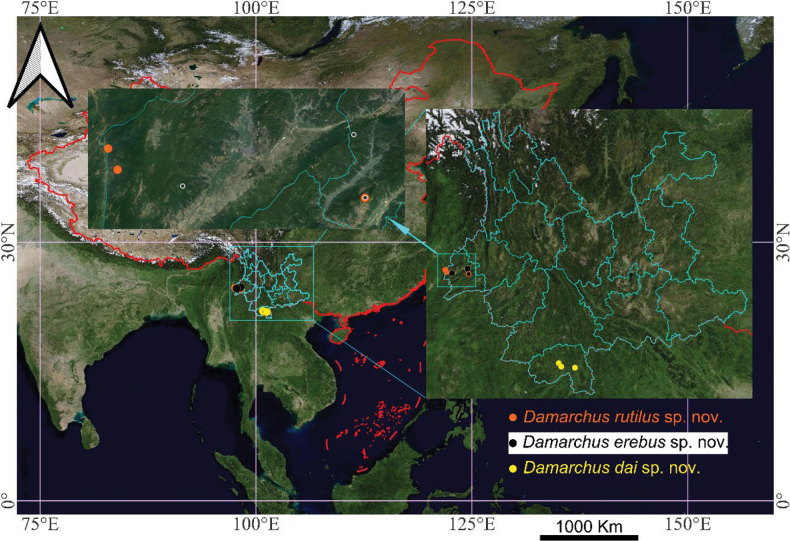
Map showing the collecting localities (●) of the three new species: *Damarchus dai* sp. nov. inhabits biotopes in southern Yunnan, while *D. erebus* sp. nov. and *D. rutilus* sp. nov. inhabit western Yunnan (type locality).

**Table 12.** *Damarchus rutilus* sp. nov., Lianghe population, female palp, and legs measurements (*n* = 1).

**Segment**	**Femur**	**Patella**	**Tibia**	**Metatarsus**	**Tarsus**	**Total**
**Appendage**						
**Palp**	3.30	1.56	2.20		2.00	**9.06**
**Leg I**	4.90	2.83	3.60	3.00	1.85	**16.18**
**Leg II**	4.43	2.70	3.10	2.75	1.72	**14.7**
**Leg III**	3.41	?	?	2.86	1.66	?
**Leg IV**	5.30	2.80	3.50	4.95	2.00	**18.55**

**Table 13.** Interspecific and intraspecific *COI* genetic distances among populations of *Damarchus dai* sp. nov., *D. erebus* sp. nov., and *D. rutilus* sp. nov. Four-digit Arabic numerals (xxxx) represent abbreviated specimen codes of KYUARA#XXXX; JH, Jinghong population; ML, Menglun population; LH, Lianghe population; YJ, Yingjiang population.

	A. 1493	B. 1494	C. 1497	D. 1498	E. 1492	F. 1524	G. 1495	H. 1496	I. 1509	J. 1514	K. 1531	L. 1540	M. 1526	N. 1507	O. 1508	P. 1530	Q. 1534	R. 1535	S. 1575	T. 1528
A. ***D. dai*** 1493 JH																				
B. ***D. dai*** 1494 JH	0.003																			
C. ***D. dai*** 1497 JH	0.044	0.048																		
D. ***D. dai*** 1498 JH	0.049	0.052	0.011																	
E. ***D. dai*** 1492 ML	0.106	0.109	0.109	0.116																
F. ***D. dai*** 1524 ML	0.111	0.111	0.114	0.122	0.019															
G. ***D. erebus*** 1495 LH	0.173	0.183	0.181	0.184	0.169	0.177														
H. ***D. erebus*** 1496 LH	0.173	0.177	0.181	0.179	0.169	0.177	0.004													
I. ***D. erebus*** 1509 LH	0.183	0.189	0.183	0.191	0.173	0.183	0.024	0.020												
J. ***D. erebus*** 1514 LH	0.183	0.190	0.183	0.189	0.173	0.183	0.023	0.018	0.006											
K. ***D. erebus*** 1531 YJ	0.197	0.203	0.203	0.205	0.185	0.189	0.139	0.139	0.143	0.141										
L. ***D. erebus*** 1540 YJ	0.195	0.195	0.201	0.205	0.181	0.183	0.136	0.136	0.133	0.133	0.003									
M. ***D. erebus*** 1526 YJ	0.197	0.197	0.200	0.205	0.181	0.187	0.138	0.138	0.134	0.134	0.027	0.027								
N. ***D. rutilus*** 1507 LH	0.154	0.157	0.164	0.177	0.138	0.141	0.166	0.164	0.170	0.172	0.177	0.168	0.178							
O. ***D. rutilus*** 1508 LH	0.154	0.157	0.164	0.179	0.138	0.141	0.168	0.166	0.174	0.170	0.179	0.168	0.178	0.004						
P. ***D. rutilus*** 1530 YJ	0.168	0.171	0.179	0.183	0.147	0.155	0.170	0.168	0.168	0.168	0.175	0.172	0.164	0.103	0.105					
Q. ***D. rutilus*** 1534 YJ	0.170	0.171	0.181	0.188	0.149	0.157	0.169	0.168	0.168	0.164	0.179	0.172	0.164	0.106	0.101	0.007				
R. ***D. rutilus*** 1535 YJ	0.175	0.177	0.189	0.192	0.158	0.166	0.177	0.175	0.177	0.174	0.177	0.170	0.170	0.113	0.108	0.028	0.024			
S. ***D. rutilus*** 1575 YJ	0.176	0.181	0.187	0.192	0.155	0.162	0.175	0.173	0.172	0.173	0.183	0.174	0.166	0.108	0.113	0.015	0.016	0.038		
T. ***D. rutilus*** 1528 YJ	0.168	0.173	0.177	0.181	0.155	0.162	0.170	0.168	0.170	0.164	0.170	0.166	0.162	0.107	0.105	0.025	0.022	0.032	0.033	

**Table 14.** Information of molecular voucher specimens and GenBank accession numbers of their COI sequences.

**Species**	**Specimen Catalog #**	**Sex**	**Location (all in Yunnan)**	**GenBank #**
*Damarchus dai*	KYUARA#1493	♂	Manmian village, Jinghong	PX967098
*Damarchus dai*	KYUARA#1494	♀	Manmian village, Jinghong	PX967104
*Damarchus dai*	KYUARA#1497	♂	Ake village, Jinghong	PX967099
*Damarchus dai*	KYUARA#1498	♀	Ake village, Jinghong	PX967105
*Damarchus dai*	KYUARA#1492	♂	Mengla County, Menglun	PX967100
*Damarchus dai*	KYUARA#1524	♀	Mengla County, Menglun	PX967101
*Damarchus erebus*	KYUARA#1495	♂	Sanjia Village, Lianghe	PX967106
*Damarchus erebus*	KYUARA#1496	♂	Zhaojiazhai, Lianghe	PX967107
*Damarchus erebus*	KYUARA#1509	♂	Kazi Village, Lianghe	PX967108
*Damarchus erebus*	KYUARA#1514	♀	Kazi Village, Lianghe	PX967109
*Damarchus rutilus*	KYUARA#1507	♂	Kazi Village, Lianghe	PX967111
*Damarchus rutilus*	KYUARA#1508	♀	Kazi Village, Lianghe	PX967112
*Damarchus erebus*	KYUARA#1531	♂	Tongbiguan, Yingjiang	PX967110
*Damarchus erebus*	KYUARA#1540	♂	Tongbiguan, Yingjiang	PX967102
*Damarchus erebus*	KYUARA#1526	♀	Provincial Road 318, Yingjiang	PX967103
*Damarchus rutilus*	KYUARA#1530	♂	Tongbiguan, Yingjiang	PX967113
*Damarchus rutilus*	KYUARA#1534	♂	Tongbiguan, Yingjiang	PX967114
*Damarchus rutilus*	KYUARA#1535	♂	Tongbiguan, Yingjiang	PX967115
*Damarchus rutilus*	KYUARA#1575	♂	Tongbiguan, Yingjiang	PX967116
*Damarchus rutilus*	KYUARA#1528	♀	Tongbiguan, Yingjiang	PX967117

Carapace (Fig. 23B) relatively smooth. With some setae on carapace, setae generally sparse along outer margin of carapace, bristles on cephalic region; those on cephalic region forming one median longitudinal row. Cephalic region obviously raised. Eyes (Fig. 23B, D) on low mound, posterior eyes noticeably whiter than anterior eyes, AME little yellow. Fovea (Fig. 23B) procurved, width ~ 1/2 width of the ocular area. Chelicerae. Paturon (Fig. 23F) with 11 promarginal teeth in one row (the eleventh tooth smallest), other teeth relatively similar in size; mesobasal denticles present adjacent to the first-third proximal teeth, numbering more than 20; dorsally bearing band of bristles that becomes broader and denser from proximal to distal end; distal bristles not forming a typical rastellum. Maxillae (Fig. 23F) possessing distinct anterior lobe and posterior heel. Cuspules on maxillae and labium peg-like, notably bigger, more distinct, stouter, and more strongly sclerotized compared to those of males; maxillae bearing cuspules as follows: KKYUARA#1528 46–55, KYUARA#1508 56–58; labium bearing 1 or 2 cuspules. Three pairs of sternal sigilla (Fig. 23I), become closer to outer margin of sternum from posterior to anterior; posterior pair distinctly larger than anterior two pairs; anterior two pairs nearly equal in size; anteriormost pair almost merging into outer margin of sternum. All sternal sigilla more sclerotized and with more defined margins compared to those of males (Fig. 23G). Opisthosoma (Fig. 23J) with four spinnerets (*n* = 2): PMS: 1.04 ± 0.08 long, 0.34 ± 0.02 diameter, PLS: 3.06 ± 0.32 long, 0.74 ± 0.05 maximal diameter, length of basal, medial, and apical segments 1.38 ± 0.18, 0.74 ± 0.08, 0.94 ± 0.06, respectively. Apical segment digitiform.

Spination of anterior legs (including large spine-like bristles; Figs 25, 30). General: Leg I, femur pd (0–0–1); tibia pd (0–0–1), rv (1–1–0); metatarsus pv (0–0–1), rv (1–1–1). Leg II, femur pd (0–0–1); patella pd (0–0–1); tibia pd (0–0–1), rv (1–1–0); metatarsus pv (0–0–1), rv (1–1–1). Variations: paratype (KYUARA#1528), rv as (1–1–1–1) on left metatarsus I, pd as (0–1–1) and pv as (1–0–1) on left tibia II. non-type (KYUARA#1508) without pd on both tibiae I, pd as (–1–1–) on both tibiae II, with additional pl (0–1–0) and v (0–1–0) on both metatarsi II.

Leg scopulae. On tarsi I–IV and distal half of metatarsi I and II.

Trichobothria. Paratype: 19–24 in two rows on tibiae, 24 in one row on metatarsi, and 11–15 in one zigzag row on tarsi. Variations: Paratype, 17 on metatarsi III; non-type, 20–22 on metatarsi I–IV.

Metatarsal preening combs. On legs II–IV; II with Pv comb only, close to base of Pv spine; III–IV with both Pv and Rv combs, close to base of Pv and Rv spines, respectively; each comb commonly composed of three teeth (short spines). Variations: 4 teeth on all combs on metatarsi II of paratype (KYUARA#1528); 2 teeth on all combs on metatarsi III of non-type (KYUARA#1508).

Claws. 2 rows of teeth, ~ 4 teeth per row on both inner and outer claws on all paired claws, proximal tooth smallest. Unpaired claw present on all tarsi and edentate, respectively. Variations: 6–7 teeth per row on outer claws and 4–5 teeth per row in non-type (KYUARA#1508).

Spermathecae (Figs 26D, 31D). Stalk long, ~ 2 × the length of receptacle, (slightly) everted, densely covered with glandular pores, broad at base, constricting markedly toward the upper 2/3; receptacle, bearing glandular pores only on uppermost surface, with the lower region devoid of pores. Variations: receptacles mushroom-shaped in paratype (KYUARA#1528) (Fig. 26D), and fan-shaped in non-type specimen (KYUARA#1508).

##### Etymology.

The specific epithet refers to the coloration (Orange-red) of the living female specimen. Noun in apposition.

##### Remarks.

All examined specimens were collected from Dehong and are considered here as belonging to two geographical populations: the Yingjiang population (type series, 4 ♂ 1 ♀ examined) and the Lianghe population (non-type specimens, 1♂ 1 ♀ examined). The *COI* genetic distance (Table 13) within the Yingjiang population ranges from 0.7–3.8% (mean distances 2.4%), while distance between the two individuals of the Lianghe population is 0.4%. A relatively significant difference is observed between the Lianghe and Yingjiang populations, with a genetic distance reaching 10.1–11.3% (mean distances ~ 10.7%). However, no reliable morphological differences were identified between these two populations based on the available specimens, particularly in the key sexual features (i.e., female vulva, male palpal organ and coupling spur). The only difference is in a paratype (KYUARA#1528), the receptacle is mushroom-shaped (Fig. 26D); in a non-type (KYUARA#1508), the receptacle is fan-shaped (Fig. 31D). This may be related to the fact that only with two female specimens were studied, and further studies will require more female specimens for comparative analysis. Given the lack of identifiable morphological distinctions, we consider it more appropriate to treat the Yingjiang and Lianghe populations as the same species in the current taxonomy of *Damarchus*, rather than splitting them based solely on the currently available molecular data.

## Discussion

Tyagi et al. (2019) examined 489 specimens from 72 genera and 21 families (Araneomorphae and Mygalomorphae) were analyzed. Genetic distances were 28.4% between suborders, 8.69–28.59% between genera, 2.11–22.30% between species (mean 9.32%), and 0–10.09% within species (mean 1.04%). High intraspecific divergence was mainly associated with potential cryptic species. After excluding these taxa, maximum intraspecific divergence decreased to 2.6%. A DNA species delimitation threshold of 2.6–3.7% is proposed for spiders.

However, several other studies have shown that intraspecific genetic divergence can be relatively large in mygalomorph spiders, which is similar to the findings of the present study: Hamilton et al. (2011) reconstructed the phylogeny of the *hentzi* species group and sister species in the North American tarantula genus, *Aphonopelma*. The greatest intraspecific divergence (5.7%) was found in the *A. hentzi* clade (specimen APH0933 from Big Bend National Park), while northern populations showed very low divergence (0.16%) and southern populations higher variation (2.6%), indicating strong geographic structure; six phylogeographic regions of *Atrax sutherlandi* Gray, 2010 occur as a linear north-south array (except for minor latitudinal overlap between regions 2 and 3) (Beavis et al. 2011). Four morphological traits display distinct and visible structural variation across the Regions (Wong et al. 2017); Monjaraz-Ruedas et al. (2023) study show between-group mean *CO I* distances range from 10.5–12.7% in the western clade, 7.8–10.5% in the *Hexurella pinea* Gertsch & Platnick, 1979 complex, and 8.5–9.6% among geographic subclades within *H. apachea*. As a point of comparison, *CO I* distances range 10–13% for hypothesized cryptic species in the atypoid mygalomorph genus *Aliatypus* (Starrett et al. 2018); Marsh et al. (2023) conducted population genetic and phylogenetic analyses of *Moggridgea rainbowi* (Pulleine, 1919) using Illumina primer sequencing of nuclear and mitochondrial sequence data. Their results revealed two populations in eastern Kangaroo Island and one population in the west that has been severely impacted by fire. High levels of genetic divergence were observed among the two populations, with differences ranging from 5% to 16%.

The three species examined in this study exhibit substantial genetic divergence in mitochondrial *CO I* sequences both among populations and, in some cases, within populations, with values generally exceeding the commonly cited 2–6% interspecific threshold. Notably, this level of genetic differentiation is not accompanied by corresponding morphological variation, as individuals from different populations remain largely indistinguishable in diagnostic morphological characters. This pronounced discordance between mitochondrial genetic distance and morphological stasis strongly argues against the reliability and utility of *CO I* barcoding as a standalone criterion for species delimitation in this group. In particular, the case of the dai Jinghong population, which spans ~ 0.3–5.2% (mean distances 3.45%), further highlights that *CO I* variation in this lineage overlaps extensively with, or even falls within, ranges typically interpreted as interspecific divergence in other taxa. Therefore, *CO I*-based thresholds appear to be inconsistent and potentially misleading in this system.

Therefore, the *CO I* sequences provided for each species in this study are intended to supplement the descriptive information of the species, rather than to serve as a basis for species delimitation or taxonomic decision-making. Further genetic studies on *Damarchus* remain to be explored.

## Supplementary Material

XML Treatment for Damarchus dai

XML Treatment for Damarchus erebus

XML Treatment for Damarchus rutilus

## Additional information

### Artificial Intelligence (AI) use

The authors accept full responsibility for the content of the manuscript, including the disclosure of any use of AI.

No AI tools were used in the preparation of this manuscript.

### Author ORCIDs

Y. Zhang https://orcid.org/0009-0007-2514-3146

K. Yu https://orcid.org/0000-0002-5517-4484

F. Zhang https://orcid.org/0000-0002-3347-1031

### Data availability

All molecular data generated in this study are deposited in GenBank under accession numbers PX967098–PX967117.

## Supplementary materials

Supplementary material 1 Raw data COI genetic distances Data type fas https://binary.pensoft.net/file/1732365This dataset is made available under the Open Database License (http://opendatacommons.org/licenses/odbl/1.0/). The Open Database License (ODbL) is a license agreement intended to allow users to freely share, modify, and use this Dataset while maintaining this same freedom for others, provided that the original source and author(s) are credited. Yunjie Zhang, Kun Yu, Feng Zhang
